# A *Lactobacillus delbrueckii* subsp. *bulgaricus* fermented skimmed milk powder has limited effects on gastrointestinal health in healthy individuals with mild-to-moderate gastrointestinal symptoms: results from a randomised controlled trial

**DOI:** 10.1017/gmb.2026.10031

**Published:** 2026-07-31

**Authors:** Martina Rooney, Clíona Ní Chonnacháin, Bernadette Finnerty, Grace Bennett, Éimear Gregory, Conor Carey, Monica Mechoud, Ram Raj Panthi, Harsh Mathur, Eoin G. Murphy, Tom Beresford, Paul D. Cotter, Alice Lucey, Emma L. Feeney

**Affiliations:** 1 University College Dublin, Food for Health Ireland, Ireland; 2 Institute of Food and Health, University College Dublin, Ireland; 3 School of Food and Nutritional Sciences, University College Cork College of Science Engineering and Food Science, Ireland; 4 Teagasc Food Research Centre Moorepark, Ireland; 5 Department of Food Biosciences, Teagasc Food Research Centre Moorepark, Ireland; 6 University College Cork APC Microbiome Institute, Ireland; 7 Vistamilk Research Centre, Teagasc Food Research Centre Moorepark, Ireland

**Keywords:** dairy, fermentate, quality of life, constipation, gastrointestinal symptoms

## Abstract

Fermented dairy foods offer potential to improve gastrointestinal symptoms due to their functional characteristics (e.g., presence of potentially health-promoting microorganisms, and/or bioactives), which may improve quality of life (QoL). This study examined the effect of a *Lactobacillus delbrueckii* subsp. *bulgaricus* fermented skimmed milk powder (FSMP) on gastrointestinal health in healthy individuals with mild-to-moderate gastrointestinal symptoms. Subjects received 20 g/day of either the FSMP, skimmed milk powder (SMP), or maltodextrin for 8 weeks. QoL, faecal calprotectin, gut transit time, gas evacuations, stool consistency, dietary intake, and anthropometry were all assessed. Eighty-four participants (62% female, 34.9 ± 5.9 years) completed the trial. SMP improved anxiety (*P* = 0.015), discomfort (*P* = 0.002), and global (*P* = 0.010) domain scores vs. maltodextrin. Similarly, SMP improved discomfort (*P* = 0.007) and global (*P* = 0.023) domain score relative to FSM*P*. No other differences between groups post-intervention were observed (*P* > 0.05). Sub-analyses indicated those with more severe symptoms at baseline had more normal stool consistency (*P* = 0.016) and fewer gas evacuations (*P* = 0.008) in response to FSM*P* relative to maltodextrin. Overall, this exploratory analysis found an *L. delbrueckii* subsp. *bulgaricus* FSMP had limited effects on gastrointestinal health in this cohort.

## Introduction

A healthy gastrointestinal environment supports nutrient digestion and absorption, and also contributes to aspects of immune, metabolic, and mental health (Bischoff, 2011). While lacking a clear definition, gut health can be referred to as a state where the gastrointestinal system functions in the absence of (i) gastrointestinal complaints (e.g., abdominal pain, bloating, diarrhoea); (ii) indications of bowel disease, for example, irritable bowel syndrome (IBS) and inflammatory bowel disease (IBD); and (iii) confirmed bowel disease (Bischoff, 2011). Gastrointestinal complaints are widely experienced in most populations, with approximately 40% of the global population experiencing ≥1 symptom associated with functional gastrointestinal disorders (FGIDs) (Sperber et al., 2021), symptom-based conditions without identifiable structural or biochemical abnormalities that explain their clinical features (Drossman and Hasler, 2016). They are characterised by symptoms such as abdominal pain and discomfort, bloating, diarrhoea, and constipation, with IBS and functional dyspepsia being the most common presentations (Drossman and Hasler, 2016), which can disrupt gastrointestinal functioning and can cause emotional distress and negatively impact quality of life (QoL) (Farndale and Roberts, 2011). FGIDs affect a substantial proportion of the population and are associated with considerable socioeconomic consequences, with the annual cost per person with IBS in Europe estimated at €3,000 (Flacco et al., 2019; Sperber et al., 2021).

FGIDs can be managed through pharmacological therapies, although lifestyle adjustments are also highly important, with a growing interest in the role of dietary intake in this context (Sayuk and Gyawali, 2015; Marasco et al., 2025). Fermented foods are of particular interest, due to their potential to positively impact the gastrointestinal environment and improve symptoms of dysfunction (Leeuwendaal et al., 2022). A variety of fermented dairy foods are commonly produced through the desirable action of microorganisms including yoghurt, kefir, fermented milk, and cheese (Marco et al., 2021). Animal and human studies show that some fermented milks, yoghurts, and kefirs can benefit those with functional gastrointestinal symptoms (Ní Chonnacháin et al., 2024). While research shows fermented dairy foods offer a potential solution to improve gastrointestinal parameters in cohorts with gastrointestinal dysfunction, the majority of studies have focused on fermented milks (liquid food matrix) and yoghurts (gel-viscoelastic food matrix), which often contain live microorganisms (Ní Chonnacháin et al., 2024). An alternative method for delivering health benefits associated with fermentation is via a dried fermented product, known as a fermentate (Mathur et al., 2020). A fermentate is typically a dried powder formulation derived from a fermented product (e.g., fermented milk) that can contain the fermenting microorganisms (e.g., lactic acid bacteria (LAB)), components of these microorganisms, culture supernatants, fermented substrates, and a range of metabolites and bioactive components with potential health benefits (Mathur et al., 2020). Dried dairy fermentates are commonly produced using LAB that undergo a heat-killing phase during processing prior to drying by either freeze-drying or spray drying, the purpose of which is to inactivate the microbes present (Mathur et al., 2020). As fresh fermented foods containing live microorganisms have a short shelf life, fermentates offer the potential for increased food safety and stability during processing, transport, and storage (Wei et al. 2024; Kaya et al. 2026), with the possibility for use as a food ingredient to enhance the nutritional profile of a variety of foods. Emerging evidence highlights the efficacy of fermentates in managing gastrointestinal disorders, reducing inflammation, and improving overall gut health (Żółkiewicz et al., 2020). Specifically, LAB have been investigated for their effects on human health, including the gut (Pasolli and Ercolini, 2020), with *Lactobacillus delbrueckii* subsp. *bulgaricus* gaining interest recently (Wang et al., 2025). Fermentation of dairy via LAB can produce bioactive substances such as peptides with angiotensin-converting enzyme-inhibitory activity (Gobbetti et al., 2000; Regazzo et al., 2010), which has been linked with inflammation and immunity (Oosthuizen and Sturrock, 2022). Cell-line work has shown *L. delbrueckii* subsp. *bulgaricus* to eliminate *Clostridium difficile* modulated cytotoxicity in addition to reducing colonisation of *C. difficile* cells in colorectal cells (Banerjee et al., 2009). Extracellular polysaccharides are produced by LAB during fermentation and have been shown to have immunomodulatory effects in mouse models (Makino et al., 2006; Nagai et al., 2011) and further confirmed in a human study to increase natural killer cell activity and reduce the risk of catching the common cold in elderly individuals (Makino et al., 2010). Trials in porcine cells suggest expression of IL-6 and proinflammatory chemokines may be implicated here (Kanmani et al., 2018).

Few studies consider the effect of fermented dairy, including fermentates, in non-clinical cohorts, despite ~40% of populations experiencing functional gastrointestinal symptoms (Sperber et al., 2021). Given the evidence base, dairy fermentates may present an accessible method of alleviating symptoms in this underserved cohort. The aim of this study was to investigate the potential of a novel dairy fermentate, made using an *L. delbrueckii* subsp*. bulgaricus* fermented skimmed milk powder (FSMP), to improve gastrointestinal health and QoL in a non-patient cohort, that is, those with self-reported gastrointestinal symptoms but without a clinical FGID diagnosis.

## Methods

### Subjects

Participants were recruited via posters and online advertisements in Dublin and Cork, Ireland, between September 2023 and April 2024. Subjects were eligible for inclusion if aged 20–45 years with self-reported mild-to-moderate digestive issues or discomfort, as assessed by a screening questionnaire comprising the Structured Assessment of Gastrointestinal Symptoms (SAGIS) questionnaire (Koloski et al., 2017), and the Discomfort domain (9 questions) of the Functional Digestive Disorders Quality of Life (FDDQoL) questionnaire (Chassany et al., 1999). Participants were included if they obtained a score of ≥11 and ≤44 in the 22-question SAGIS questionnaire, and >13.5 and ≤36 in the FDDQoL Discomfort domain, indicating a score between mild and moderate symptoms in 50%–100% of questions. In line with established clinical cut-off points for anxiety and depression (Hansson et al., 2009), those with scores of ≤10 in these Hospital Anxiety and Depression Scale (Zigmond and Snaith, 1983) domains were excluded. The cut-offs were chosen to ensure the cohort represented “healthy” individuals, meaning participants did not require clinical intervention for gastrointestinal or mental health conditions due to severe gut–brain axis disruptions (Rogers et al., 2016). Exclusion criteria included lactose intolerance, very mild or very severe gastrointestinal issues, post-menopausal females or those experiencing menopausal symptoms, being prescribed antibiotics, medications for gastrointestinal issues or probiotics within the previous three months, medical issues affecting gut transit/digestion, smoking, regular antacid use, pregnant or lactating, not living independently, or diagnosis of type 1 diabetes, IBD, or IBS. All study procedures were approved by the University College Dublin Human Research Ethics Committee (LS-23-20-Feeney) and Clinical Research Ethics Committee of the Cork Teaching Hospitals (ECM 3 (i) 12/09/2023), and written, informed consent was obtained from each participant prior to commencing the study. The study is registered as ISRCTN17696788.

### Study design

This multicentre study was a double-blind, parallel-arm RCT with three intervention arms, conducted at University College Dublin (UCD) and University College Cork (UCC) in Ireland. The overall study duration was 16 weeks. Phase 1 was a 4-week baseline period to establish patterns of gut comfort/discomfort. Phase 2 was an 8-week intervention phase where participants were randomised to one of three treatment arms. Phase 3 was a 4-week “washout” period to determine the persistence of treatment effect. In order to further explore the mechanism behind any potential effects of an *L. delbrueckii* subsp. *bulgaricus* fermented dairy powder on gut health, two comparators were employed. A non-fermented SMP was used to isolate any fermentation-specific effects, whilst an energy-matched, non-dairy maltodextrin control allowed isolation of any dairy-specific effects. Allocation to FSMP, SMP, or maltodextrin was on a 3:1:1 (Dumville et al., 2006) basis. The unbalanced design was chosen due to the perceived health benefits of the FSMP based on previous literature highlighting the potential of LAB fermentates to benefit gastrointestinal parameters (Tarrerias et al., 2011; Sawada et al., 2016). A statistician was consulted regarding this approach (Dumville et al., 2006), who advised that equal sample size is not an assumption for ANOVA. Any baseline variability is addressed by multiple measurements pre-intervention for baseline phenotyping, as described later. Block randomisation was performed by an independent person not associated with the study, to assign subjects to one of five treatments codes, which ensured both participants and researchers assessing the outcome measurements were fully blinded given the 3:1:1 allocation.

### Intervention foods

The intervention arm was an FSMP, SMP was an active control, and maltodextrin was a non-dairy placebo. SMP was chosen to compare the effect of fermentation on gut health, whilst maltodextrin, an energy-matched, non-dairy comparator, enables elucidation of any dairy-specific effects. The daily dose was 20 g test powder mixed in 200 mL water, for which a beaker with shaker was provided. FSMP and SMP were supplied by Teagasc Food Research Centre, Moorepark, Cork, Ireland. Maltodextrin (Maldex® 190) was supplied by Carbery Group Ltd, Cork, Ireland. FSMP was fermented with *L. delbrueckii* subsp. *bulgaricus.*

FSMP was prepared as follows: SMP (Dairygold, Cork, Ireland) was entrained in water at 50 °C (25% w/w; total weight – 1000 kg) using a vortex-inducing high-shear impellor system (APV Crepaco, WI, USA). The reconstituted skim milk was then heat-treated at 115 °C for 29 s (APV/SPX, NC, USA) and subsequently cooled to approximately 50 °C and transferred to a fermentation tank, where it was allowed to reach the target fermentation temperature of 37 °C. The inoculation medium was added at a rate of 1% (w/w), and the fermentation was allowed to proceed for 24 h at 37 ± 1 °C. After 24 h, the pH of the fermentate was re-adjusted to its starting pH using a 50:50 mixture of 1 M NaOH and 1 M KOH. The fermentate live culture was then inactivated by heat treatment (80 × 29 s; APV/SPX, NC, USA). Finally, the fermentate was dried using a tall-form spray dryer (GEA-Niro, Søborg, Denmark).

Several LAB species were screened for their ability to efficiently ferment a 10% w/v reconstituted skim milk substrate at laboratory scale. Selection of *L. delbrueckii* subsp. *bulgaricus* was based on quantifying increases in counts of colony-forming units/mL, pH reductions, conversion of lactose to lactic acid, reduction in the concentration of lactose, and release of free amino acids. Non-fermented samples were subjected to the same heat treatment served as the active control (SMP). The macronutrient content of each study treatment is provided in Supplementary Table 1. Energy and fat content were similar across the three treatment powders. Both FSMP and SMP contained higher amounts of carbohydrate and protein than the maltodextrin. The organic acid composition of the dairy-based test powders is presented in Supplementary Figure 1. Lactic acid, galactose, and glucose were present in FSMP but not in SMP, while higher concentrations of lactose were present in SMP compared to FSMP. All test powders were provided in identical 400 g aluminium cans. Compliance was assessed by weighing the cans of powder at the start and end of the intervention period. Subjects were advised to maintain their normal diet. Participants were blinded to any differences between test powders owing to the parallel-arm study design, while researchers were blinded as the cans were sealed at all times.

### Measurements

**Figure 1.**
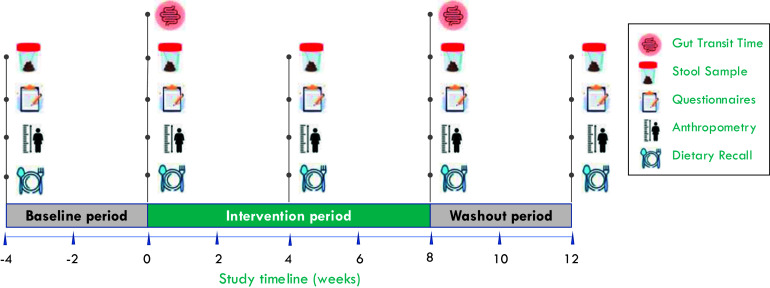
Study design: Diagrammatic representation of 16-week trial with three phases: 4-week baseline period, 8-week intervention phase, a 4-week “washout” period. During the intervention period, participants consumed 20 g per day of an FSMP, SMP, or maltodextrin. Diagram shows the five time points for participant study visits, data collection, and biological sample collection. Figure 1 long description.

The study protocol is outlined in Figure 1. Participants attended the Human Intervention Study Suite at the Institute of Food and Health in UCD, or the Human Nutrition Studies Unit at the School of Food and Nutritional Sciences in UCC, on five occasions, that is, Week −4 (run-in), Week 0 (pre-intervention), Week 4 (mid-point), Week 8 (post-intervention), and Week 12 (persistence).

### Anthropometry

Anthropometric measurements were obtained at each visit, in duplicate, according to standard operating procedures. Body weight was measured on a Tanita (Model BC-420MA, UCD) or SECA (SECA 803 weighing scales, UCC) scale. Height was measured with a freestanding stadiometer (Leicester portable height measures, Chasmores Ltd., UK).

### Questionnaires

The primary outcome of the study was the effect of treatment on QoL, as assessed by the FDDQoL questionnaire, measured weekly throughout the 16-week study period (Chassany et al., 1999). The Perceived Stress Questionnaire (PSQ) was also administered weekly (Levenstein et al., 1993). The remaining questionnaires were administered at each study visit: Subject’s Global Assessment of Relief (SGA) (Müller-Lissner et al., 2003), Patient Assessment of Constipation Quality of Life (PACQoL) questionnaire (Marquis et al., 2005), and the Bristol Stool Form Scale (Lewis and Heaton, 1997). Gas evacuation logs were completed in the 3 days preceding each visit, except for the initial visit at Week −4. Dietary intake was assessed by a 24-hour dietary recall at each study visit, administered using the web-based dietary assessment method, Foodbook24 (Timon et al., 2017).

### Gut transit time (GTT)

GTT was assessed at Week 0 and Week 8, that is, pre- and post-intervention, using the blue dye method (Asnicar et al., 2021). Two “blue” muffins containing royal blue food colouring paste were consumed within a 10-minute period. Subjects logged the first appearance of “blue poo” via an online questionnaire. GTT was measured in hours and minutes, from the time of muffin consumption to the first visualisation of blue within a stool (Asnicar et al., 2021). GTT was measured in *n* 66 participants; *n* 1 participant did not consume the muffins due to a food allergy, *n* 6 did not measure GTT as blue was not observed in their stool following muffin consumption, and *n* 11 did not submit a report on at least one occasion.

### Stool samples (faecal calprotectin)

Stool samples were collected on the morning of study appointments and stored in a cooler bag with ice packs until arrival at the test centre. Samples were refrigerated immediately upon arrival and processed on the same day. A 10 g portion of sample from Week −4 (run-in), Week 4 (mid-point), and Week 12 (persistence) was sent to Eurofins Biomnis (Dublin, Ireland) for faecal calprotectin (FCP) analysis. Calprotectin testing was performed on the Phadia 250 instrument, using the Phadia EliA method (Thermo Fisher Scientific, Uppsala, Sweden). Remaining sample was stored at −80 ^o^C.

### Statistical analysis

Statistical analysis was conducted using SPSS, V29.0.1.0 (SPSS Inc., Chicago, IL, USA). Descriptive statistics were performed on demographic data, including anthropometry, questionnaire, and dietary intake data at each time point, for the total population and stratified by treatment group. Normality of distribution and equal variance were examined using visual assessment of plots and Levene’s test of normality. Differences in categorical values were examined using χ^2^ testing. For numerical variables, differences in values at each time point were tested via 1-factor ANOVA or Kruskal–Wallis nonparametric ANOVA where appropriate. Delta (Δ) scores were calculated by subtracting the Week 0 values from the Week 8 values. Within-group differences were calculated with paired-samples T-Test. General linear models were used to evaluate differences across treatment groups, adjusting for study site, sex, age, BMI, and values at Week 0 with *post hoc* analysis *via* LSD test. Values are presented as mean ± SD, as medians and interquartile ranges (IQR), or as numerical value, *n*, and percentage, where appropriate. Dietary intake data are reported as a percentage of total energy (%TE) or grams per day, as appropriate. Results are presented in graphical and tabular format, and values of *P* < 0.05 were considered statistically significant. The primary outcome was a comparison of change in self-reported QoL, as assessed by the FDDQoL questionnaire from Week 0 in response to 8-week intervention with a fermented and non-fermented SMP or control powder. As an exploratory trial, a power calculation was not performed, although a target sample size of 120 participants was set. This is similar to an RCT by Pinheiro et al. (2017) who investigated the effect of a yeast fermentate in 80 subjects with gastrointestinal symptoms, and stratified the intervention groups by symptom severity with sample sizes ranging from 12 to 40 participants.

The present analysis considers the effects of the test powders in response to intervention, that is, phase two of the study (Week 0 vs. Week 8). Data from phase 1 (Week −4 to Week 0) were analysed to determine symptom severity at baseline for stratification analysis. Data from phase 3 (Week 8 to Week 12) were intended to investigate the persistence of any effect observed; however, this was not performed owing to the limited intervention results. This analysis presents those who completed the intervention, as is recommended for nutritional intervention studies (Welch et al., 2011) and in line with previous RCTs (Feeney et al., 2018; O’Connor et al., 2024; Rooney et al., 2025a, 2025b, 2025c; Ní Chonnacháin et al., 2026).

Two sub-group analyses were performed. First, the effect of gender on the effect of treatment on QoL was investigated, since LAB may exhibit gender-dependent effects (Yurkovetskiy et al., 2013) which has the potential to influence the results. Second, the effect of symptom severity at baseline on response to intervention was explored, as per an approach taken by Pinheiro et al. in a similar study in those with moderate-to-severe symptoms of gastrointestinal discomfort and constipation (Pinheiro et al., 2017). Baseline severity scores were calculated as the mean of FDDQoL Global domain scores from questionnaires completed in the run-in period. The mean score was 59.1 ± 11.1, with those scoring below this classified as severe, while those scoring above classified as moderate.

## Results

### Study participants and compliance

**Figure 2.**
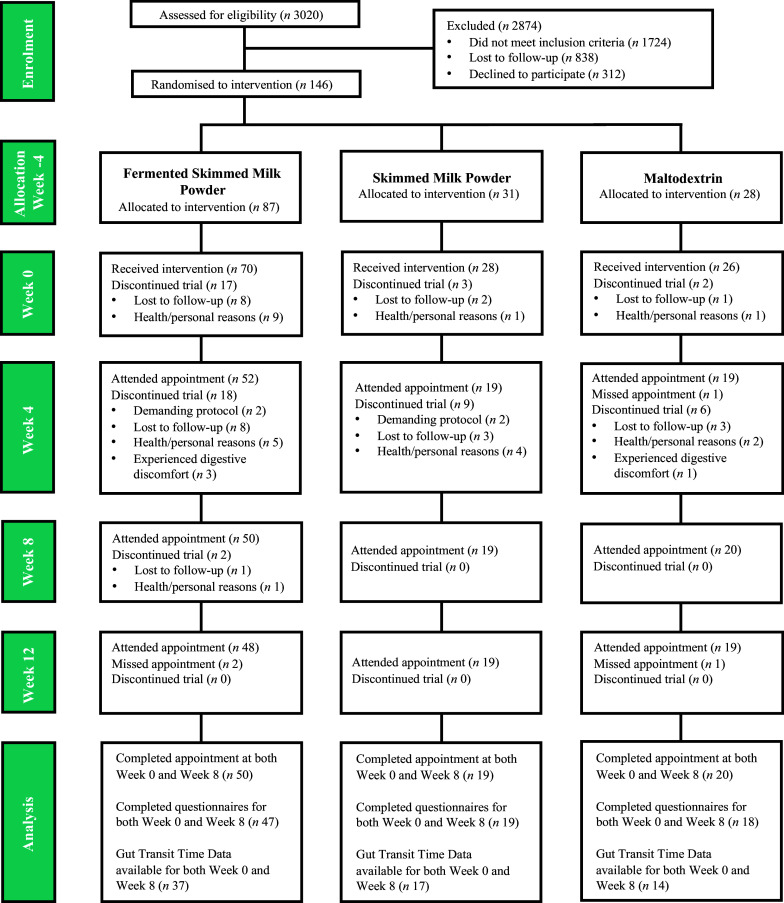
CONSORT flow diagram of participants through the study protocol. Figure 2 long description.

The flow of participants is presented in Figure 2. A total of 3,020 people completed the screening questionnaire and *n* 146 agreed to participate in the study and were randomised to intervention, *n* 106 in UCD and *n* 40 in UCC. At Week 0, *n* 124 attended a study visit, with *n* 22 discontinuing the trial owing to health/personal reasons (*n* 11) or being lost to follow-up (*n* 11). At Week 4, *n* 90 participants completed the mid-way assessment, while *n* 1 did not attend this visit, but completed all other appointments and is included in this analysis. At this mid-point, *n* 33 discontinued the trial, *n* 11 for health/personal reasons, *n* 4 due to demanding study protocol, *n* 4 for experiencing digestive discomfort, and *n* 14 were lost to follow-up at this point. At Week 8, *n* 89 attended the post-intervention appointment, with *n* 1 withdrawing for personal/health reasons and *n* 1 lost to follow-up. These 89 participants were regarded as study completers. Follow-up data at Week 12 are available for *n* 86 participants. Rates of attrition were similar for FSMP (28.6%), SMP (32.1%), and maltodextrin (26.9%). The reasons for discontinuing the trial are similar across treatment groups.

Data in the current analysis are presented for the *n* 84 participants who have completed Week 0 and Week 8 data for FDDQoL and PSQ. Based on the weight of returned powder, compliance for the cohort as a whole was 89%, indicating a high level of adherence to the protocol.

### Demographics and anthropometry

The overall mean ± SD age was 34.9 ± 5.9 years, and BMI was 26.2 ± 4.4 kg/m^2^, indicating a young adult poplation with overweight. The cohort was 38.1% (*n* 32) male and 61.9% (*n* 52) female. There were no significant differences in demographic or anthropometric variables between the intervention groups at Week 0 or in response to intervention (Table 1). No differences were observed in demographics between study sites (Supplementary Table 2).

**Table 1.** Demographics, anthropometry, and functional digestive disorders quality of life (FDDQoL) scores throughout the study protocol (*n* 84) Table 1. long description.

	Total(*n* 84)	FSMP(*n* 47)	SMP(*n* 19)	Maltodextrin (*n* 18)	*P*Wk 0^a^	*P* Δ^b^
**Demographics and anthropometry**						
Age (years)	34.9 ± 5.9	35.6 ± 6.4	34.0 ± 5.1	34.2 ± 5.3	0.575	-
**Sex** ^c^ ***n* (%)**					0.821	-
Male	32 (38.1)	17 (36.2)	7 (36.8)	8 (44.4)		
Female	52 (61.9)	30 (63.8)	12 (63.2)	10 (55.6)		
**Weight (kg)**					0.636	0.867
Week 0	77.6 ± 16.2	78.2 ± 14.7	76.8 ± 20.7	77.1 ± 15.3		
Week 8	77.7 ± 16.1	78.3 ± 14.7	76.6 ± 20.3	77.1 ± 15.4		
**BMI (kg m**^**2**^**)**					0.411	0.911
Week 0	26.2 ± 4.4	26.6 ± 4.5	26.0 ± 5.1	25.2 ± 3.6		
Week 8	26.2 ± 4.5	26.7 ± 4.5	26.0 ± 5.2	25.2 ± 3.8		
**FDDQoL**						
**Daily activities**					0.410	0.393
Week 0	77.4 ± 18.7	75.0 ± 19.6	78.7 ± 18.8	82.4 ± 15.6		
Week 8	86.3 ± 15.4	84.8 ± 17.2	90.5 ± 12.1	85.9 ± 13.4		
Δ	8.9 ± 17.1	9.8 ± 18.1	11.8 ± 16.2	3.5 ± 15.1		
*P* within group^c^		<0.001	0.006	0.341		
**Anxiety**					0.827	0.045^e^
Week 0	60.5 ± 24.6	60.0 ± 24.4	58.9 ± 28.7	63.6 ± 21.3		
Week 8	67.6 ± 21.0	66.5 ± 21.9	73.5 ± 17.5	64.5 ± 21.8		
Δ	7.1 ± 17.7	6.5 ± 15.3	14.6 ± 20.8	0.9 ± 18.3		
*P* within group^c^		0.006	0.007	0.836		
**Diet**					0.304	0.672
Week 0	56.1 ± 22.6	53.9 ± 23.0	63.2 ± 22.1	54.5 ± 21.7		
Week 8	63.8 ± 22.5	62.3 ± 22.5	71.3 ± 18.3	59.9 ± 25.7		
Δ	7.7 ± 14.7	8.4 ± 11.6	8.1 ± 18.9	5.4 ± 17.7		
*P* within group^c^		<0.001	0.077	0.215		
**Sleep**					0.809	0.376
Week 0	79.3 ± 18.2	79.4 ± 18.5	76.3 ± 21.2	81.9 ± 14.1		
Week 8	82.0 ± 16.3	80.3 ± 15.8	82.5 ± 19.4	86.1 ± 14.0		
Δ	2.7 ± 15.5	0.9 ± 12.7	6.2 ± 19.6	4.2 ± 17.4		
*P* within group^c^		0.634	0.189	0.325		
**Discomfort**					0.936	0.005^f^
Week 0	53.5 ± 18.2	53.2 ± 18.6	54.8 ± 17.4	52.9 ± 19.0		
Week 8	60.7 ± 18.9	59.5 ± 18.5	70.3 ± 15.4	53.9 ± 20.1		
Δ	7.2 ± 15.9	6.3 ± 14.7	15.5 ± 18.3	1.0 ± 13.5		
*P* within group^c^		0.005	0.002	0.757		
**Health perceptions**					0.740	0.435
Week 0	57.0 ± 10.8	56.6 ± 11.1	57.2 ± 12.4	57.9 ± 8.7		
Week 8	54.2 ± 9.6	53.6 ± 9.9	56.2 ± 9.7	53.7 ± 8.9		
Δ	−2.8 ± 11.6	−3.0 ± 11.9	−1.0 ± 13.3	−4.2 ± 9.2		
*P* within group^c^		0.089	0.734	0.070		
**Coping with disease**					0.697	0.513
Week 0	53.2 ± 22.6	53.5 ± 23.0	56.1 ± 19.2	49.1 ± 25.5		
Week 8	54.5 ± 23.6	53.5 ± 23.6	56.6 ± 22.3	54.6 ± 25.9		
Δ	1.3 ± 15.1	0.0 ± 16.3	0.5 ± 13.5	5.5 ± 13.1		
*P* within group^c^		1.000	0.888	0.090		
**Impact of stress**					0.325	0.591
Week 0	47.4 ± 22.9	45.4 ± 24.2	54.4 ± 22.5	45.4 ± 19.2		
Week 8^d^	48.4 ± 24.0	48.9 ± 24.8	52.6 ± 28.1	42.6 ± 16.6		
Δ	1.0 ± 16.9	3.5 ± 17.1	−1.8 ± 17.3	−2.8 ± 15.7		
*P* within group^c^		0.257	0.663	0.462		
**Global score**					0.722	0.024^g^
Week 0	61.0 ± 14.0	60.0 ± 14.4	62.9 ± 15.0	61.8 ± 12.1		
Week 8	66.0 ± 12.5	64.8 ± 12.3	71.3 ± 11.7	63.4 ± 13.0		
Δ	5.0 ± 9.2	4.8 ± 8.7	8.4 ± 10.7	1.6 ± 8.1		
*P* within group^c^		<0.001	0.003	0.410		

Data presented as mean ± standard deviation. A higher FDDQoL score is associated with a better quality of life. Abbreviations: Δ, delta values calculated as Week 8 minus Week 0; FSMP, fermented skimmed milk powder; SMP, skimmed milk powder.

a Differences across groups for Week 0 calculated with 1-factor ANOVA or Kruskal–Wallis nonparametric ANOVA where appropriate.

b Differences across groups for delta values (Week 8 – Week 0) calculated with general linear models controlling for study site, sex, age, BMI, and Week 0 values. *Post hoc* analysis was conducted *via* LSD test.

c Within-group differences calculated with paired-samples T-Test.

d Total *n* 83, fermented skimmed milk powder *n* 46 for Week 8 impact of stress.

e Significant differences for Δ anxiety score between SMP and Maltodextrin (*P* = 0.015).

f Significant differences for Δ discomfort score between FSMP group and SMP (*P* = 0.007) and between SMP and Maltodextrin (*P* = 0.002).

g Significant differences for Δ global score between FSMP and SMP (*P* = 0.023) and between SMP and Maltodextrin (*P* = 0.010).

### Functional digestive disorders quality of life

**Figure 3.**
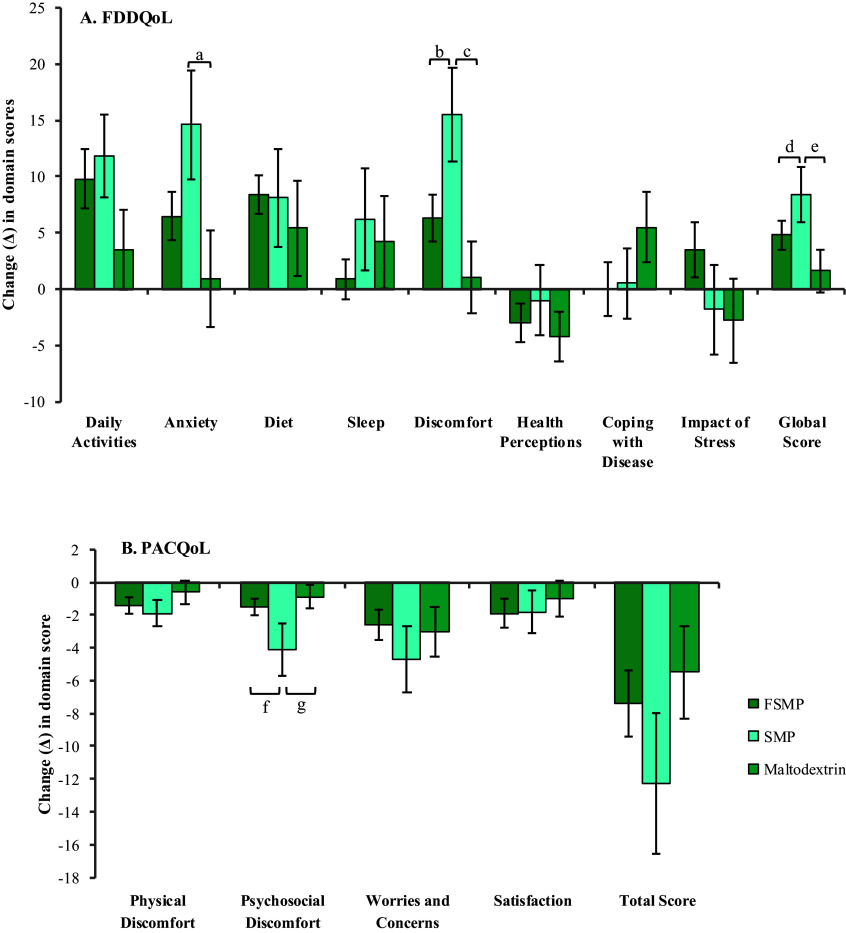
Change (Δ) in FDDQoL (A) and PACQoL (B) domain scores across treatment groups between Week 0 and Week 8. Data presented as change (Δ) in domain score between Week 0 and Week 8. A higher FDDQoL score is associated with a better QoL. A lower PACQoL score is associated with a better QoL. Abbreviations: *, P < 0.05. ^a^Significant differences for Δ anxiety score between SMP and Maltodextrin (P = 0.015). ^b^Significant differences for Δ discomfort score between FSMP group and SMP (P = 0.007). ^c^Significant differences for Δ discomfort score between SMP and Maltodextrin (P = 0.002). ^d^Significant differences for Δ global score between FSMP and SMP (P = 0.023). ^e^Significant differences for Δ global score between SMP and Maltodextrin (P = 0.010). ^f^Significant differences for Δ psychosocial discomfort between FSMP group and SMP (P = 0.031). ^g^Significant differences for Δ psychosocial discomfort between SMP and Maltodextrin (P = 0.019). Figure 3 long description.

FDDQoL is stratified into nine domains, with higher scores indicating a better QoL. Global domain score for the cohort as a whole was 61.0 ± 14.0 at Week 0, with no differences between groups (Table 1), similar to published values in a similar population (Guyonnet et al., 2007). After the 8-week intervention period, there was an increase in anxiety domain score (partial *η*^2^ = 0.053), indicating an improvement in anxiety, in response to SMP compared to maltodextrin (10.3, 95% CI [−3.6, 24.2], *P* = 0.015). SMP was found to increase discomfort domain score, indicating an improvement (partial *η*^2^ = 0.131) compared to both FSMP (11.3, 95% CI [1.1, 21.5], *P* = 0.007) and maltodextrin (14.7, 95% CI [2.9, 26.6], *P* = 0.002). Similarly, global domain score was shown to significantly increase (partial *η*^2^ = 0.065), indicating an improvement, in response to SMP compared to FSMP (4.1, 95% CI [1.8, 10.0], *P* = 0.023) and maltodextrin (5.9, 95% CI [0.9, 12.7], *P* = 0.010) after 8 weeks (Figure 3A).

### Perceived stress questionnaire

The PSQ is stratified into eight domains, with lower scores indicating improved QoL. At Week 0, the mean ± SD PSQ index score was 0.41 ± 0.15 (Supplementary Table 3). Some differences were observed between groups at Week 0, with those allocated to FSMP reporting higher irritability scores compared to maltodextrin (*P* = 0.024) and higher scores for lack of joy (*P* = 0.014), fatigue (*P* = 0.024), and worries (*P* = 0.003) relative to those allocated to SMP. No differences across groups were observed in response to 8-week intervention.

### Patient assessment of constipation quality of life

PACQoL is stratified into five domains, where lower scores indicate a better QoL. Total score domain was 42.1 ± 22.0 at Week 0 for the cohort as a whole (Supplementary Table 4). After the 8-week intervention, psychosocial discomfort score decreased (partial *η*^2^ = 0.109), indicating an improvement, in response to SMP compared to FSMP (−2.5, 95% CI [−5.5, 0.4], *P* = 0.031) and maltodextrin (−3.1, 95% CI [−6.5, 0.2], *P* = 0.019). No other differences in PACQoL domain scores were observed.

### Subject global assessment of relief

SGA comprises one question, with a higher score indicating better QoL. At Week 0, the mean ± SD SGA score was 2.3 ± 0.6. No differences at Week 0, or in response to intervention, were noted (Supplementary Table 5).

### Bristol stool form chart (BSFC)

Complete BSFC data were available for *n* 63. Mean ± SD score was 3.7 ± 1.4 at Week 0 for the cohort as a whole, indicating a normal stool consistency (Lewis and Heaton, 1997). There were no differences between groups at Week 0 or in response to intervention (Supplementary Table 5).

### Gas evacuations

Daily gas evacuation data were available for *n* 58. At Week 0, mean ± SD daily count for the cohort as a whole were 14.6 ± 10.4 evacuations. No differences between or within groups were observed (Supplementary Table 5).

### Gut transit time

**Figure 4.**
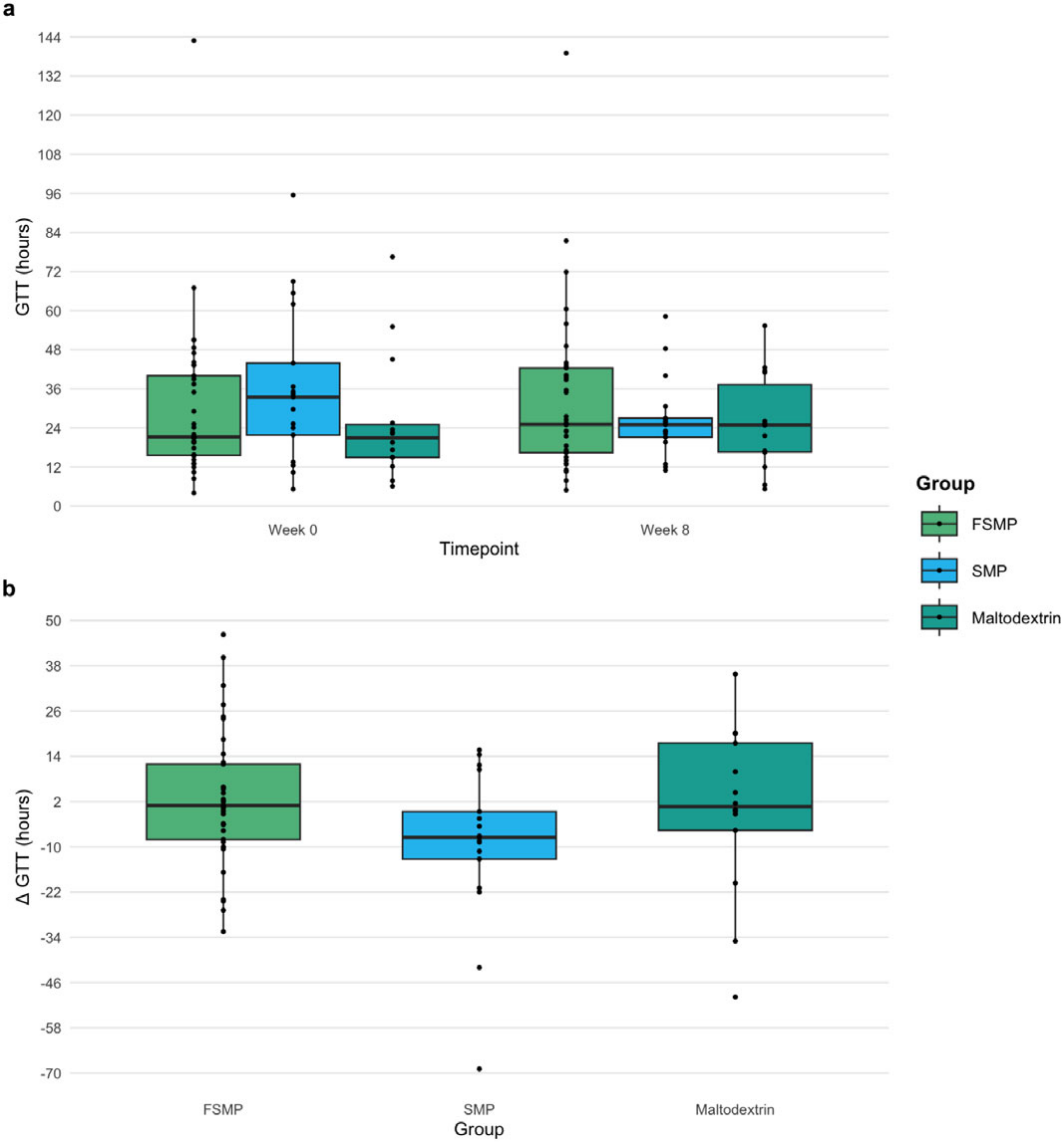
Gastrointestinal transit time (GTT) across intervention group and study period (n 68), assessed by “blue dye” method (Asnicar et al., 2021). Gastrointestinal transit time in hours split by treatment group and timepoint (A) and Change (Δ) in gastrointestinal transit time in hours across treatment groups between Week 0 and Week 8 (B). Boxes represent interquartile ranges (Q3–Q1) and horizontal lines within boxes represent medians. GTT, gastrointestinal transit time; FSMP, fermented skimmed milk powder; SMP, skimmed milk powder. Figure 4 long description.

Median GTT across all intervention groups (*n* 68) at Week 0 was 22 hours, 27 minutes (IQR: 15:30–42:26). This is faster than a previous study using the blue dye method which reported a median GTT of 28 hours, 42 minutes in healthy participants (Asnicar et al., 2021). No differences between groups at Week 0 or in response to intervention were observed (Supplementary Table 6, Figure 4).

### Faecal calprotectin

Participants with FCP <150 μg/g were included in the FCP analysis (*n* 72), in line with STRIDE II guideline thresholds (Turner et al., 2021). At Week −4, median FCP was 10.00 μg/g (IQR: 4.00–27.75). No differences between or within groups were noted (Supplementary Table 7, Supplementary Figure 2).

### Dietary intake data

At Week 0, mean ± SD energy intake was 1787 ± 604 kcal/day (Supplementary Table 8). Macronutrient intake, expressed as a %TE, was 17.3 ± 5.5% from protein, 38.2 ± 8.4% from fat, and 45.7 ± 9.3% from carbohydrate. Mean fibre intake was 15.9 ± 8.3 g/day. After the 8-week intervention period, fat intake increased in the SMP compared to FSMP group (6.9 ± 3.6%TE, *P* = 0.005). No other differences in dietary intake were observed during the study period.

### Sub-group analysis based on gender

Results for QoL indicators for males and females are presented in Supplementary Tables 9 and 10. No effects were observed within males only. However, within females SMP improved FDDQoL global domain score to a greater extent than maltodextrin (*P* = 0.010). No other differences were observed.

### Sub-group analysis based on symptom severity

Results from outcome measures stratified by symptom severity are presented in Supplementary Tables 11–13. Within those experiencing severe symptoms (*n* 42), an improvement in constipation, assessed by PACQoL score, partial *η*^2^ = 0.323, in response to SMP compared to FSMP (−18.8, 95% CI [−34.5, −3.0], *P* = 0.007) and maltodextrin (−19.1, 95% CI [−34.5, 0.1], *P* = 0.014) was found. For PSQ index score, a large effect size was observed partial *η*^2^ = 0.160, SMP was shown to improve PSQ index score relative to maltodextrin (0.37, 95% CI [0.01, 0.25], *P* = 0.017), indicating a reduction in perceived stress. A large effect size was observed for BSFC, partial *η*^2^ = 0.255, where FSMP increased stool consistency scores compared to maltodextrin (1.3, 95% CI[0, 2.7], *P* = 0.016), with those in the FSMP group maintaining a normal consistency, and those in the maltodextrin group moving into the harder stool consistency category in response to intervention. Similarly, a large effect size on daily gas evacuations was observed (partial *η*^2^ = 0.313), with a significantly decrease in response to FSMP compared to SMP (−24.1, 95% CI [−45.9, −2.3], *P* = 0.008). No other differences in total domain scores in response to treatment between groups were observed within the sub-group experiencing severe symptoms. For those experiencing moderate symptoms (*n* 42), a significantly higher GTT was observed at Week 0 within the SMP group compared to the maltodextrin group (*P* = 0.006) with a mean difference of 25 hours and 18 minutes. No differences in response to intervention were observed.

## Discussion

This study found a novel dairy fermentate, an *L. delbrueckii* subsp. *Bulgaricus* FSMP failed to affect gastrointestinal health in young adults with self-reported gastrointestinal symptoms, compared to a non-fermented SMP and energy-matched, non-dairy maltodextrin. However, non-fermented SMP was shown to improve several QoL outcomes, with improved scores observed for anxiety, discomfort, psychosocial discomfort, and global domain scores compared to both the dairy fermentate (FSMP) and the maltodextrin control. While those with more severe symptoms at baseline did appear to have improved stool consistency and reduced gas evacuations in response to FSMP, the exploratory nature and small sample size limit the application of the findings. Overall, a non-fermented SMP was found to have some beneficial effects on QoL and gastrointestinal health in those with self-reported symptoms; however, the novel, FSMP failed to elicit an effect in the population studied.

The European Medicines Agency identifies health-related QoL as the most important secondary endpoint in clinical trials assessing therapies for IBS (European Medicines Agency, 2014). Thus, several published RCTs examining the effect of fermented dairy products on cohorts with FGIDs have used QoL markers as a primary outcome, as they help to assess the extent to which symptoms may impact the daily lives of patients (Van Zanten et al., 1999; Simrén et al., 2010; Šmid et al., 2016; Gomi et al., 2018). However, evidence of significant improvements in these outcomes in response to interventions has been limited and inconsistent (Simrén et al., 2010; Šmid et al., 2016; Gomi et al., 2018), perhaps owing to the subjective nature and inter-individual variation in personal perceptions of QoL (Lackner et al., 2011). Guyonnet et al. explored the effects of a fermented milk product containing *Bifidobacterium animalis* DN-173 010 versus a non-fermented yoghurt product on QoL markers in 267 constipation-predominant, largely female IBS patients aged 20–65 years (Guyonnet et al., 2007). In that 6-week multicentre RCT, no significant differences were observed between the groups across several FDDQoL domains. Similar to the current study, Guyonnet et al. (2007) recruited participants characterised as having mild-to-moderate gastrointestinal symptoms. It is worth noting that baseline FDDQoL status in the Guyonnet et al. study was similar to the cohort in the current RCT, indicating that participants had a relatively high QoL as baseline (Guyonnet et al., 2007). Initial severity of IBS symptoms has previously been reported to significantly affect the proportion of patients who experience symptom and QoL improvements (Whitehead et al., 2006). Individuals with severe starting symptoms tend to experience greater objective improvements in symptom severity and QoL but are less likely to report satisfactory relief, while those with mild symptoms often report higher levels of satisfaction despite showing limited measurable improvement in IBS QoL (Van Zanten et al., 1999; Whitehead et al., 2006). This highlights the challenge of demonstrating treatment effects in populations with mild-to-moderate symptoms, particularly when self-reported QoL is already relatively high. The baseline scores observed in the current study population suggest that this cohort had relatively mild gastrointestinal symptoms, reducing the potential to detect measurable improvements in QoL outcomes and may have limited any detectable findings.

No effect was observed in PACQoL scores or stool consistency, measured by BSFC, in response to any of the dietary interventions in the present trial. Pinheiro et al. investigated the effects of a dried yeast fermentate (EpiCor®) in a healthy adult population aged 20–69 years using similar gastrointestinal health markers, including PACQoL and the BSFC. Although no differences were observed between the treatment and control groups (Pinheiro et al., 2017), within-group analysis revealed significant improvements in QoL in both the treatment and control groups, suggesting a potential placebo effect. In sub-group analyses, when participants were stratified according to symptom severity, the moderate sub-group appeared less affected by this placebo effect. Pinheiro et al. proposed that individuals experiencing symptoms that are more pronounced, like those in the severe group, may be more susceptible to reporting subjective improvements, highlighting the key effect that baseline severity has on reported outcomes (Whitehead et al., 2006; Pinheiro et al., 2017). The present study followed a similar stratification protocol, where SMP was shown to have more favourable outcomes with respect to both PSQ and PACQoL relative to the other interventions, only within those experiencing more severe symptoms, further emphasising the important role that initial degree of symptom severity plays in determining treatment outcomes. The inter-individual variation demonstrated here provides evidence for personalised nutrition, although the exploratory nature and small sample size here limits the strength of application of these findings.

Previous research has shown that certain fermented dairy products can significantly reduce GTT, albeit in cohorts with GI dysfunction characterised by constipation (Agrawal et al., 2009; Waller et al., 2011; Dimidi et al., 2014). Agrawal et al. showed consumption of a fermented milk product containing *Bifidobacterium lactis* DN-173 010 reduced colonic and orocaecal transit times relative to a non-fermented dairy product in 34 female constipation-dominant IBS patients (Agrawal et al., 2009). Similarly, a cross-over RCT conducted by Malpeli et al. demonstrated that consumption of a synbiotic yoghurt containing *Bifidobacterium* BB12*, Lacticaseibacillus casei* CRL 431, and fibre significantly reduced intestinal transit time compared to a control yoghurt in 83 healthy women with functional constipation (Malpeli et al., 2012). These results contrast with the findings in the current study, as neither the fermented nor the non-fermented SMP had any effect on GTT. However, the previous studies exhibiting a treatment effect focused on individuals with constipation, whereas the current study included participants with a variety of functional gastrointestinal symptoms, capturing both constipation and diarrhoea. Compared to previous findings, this cohort reported considerably shorter GTT compared to other studies in cohorts with functional constipation (Agrawal et al., 2009; Waller et al., 2011). The inclusion of a heterogeneous cohort here may have also limited the ability to detect improvements in constipation-specific outcomes such as PACQoL, stool consistency, and GTT. Given that the strongest effects tend to be observed in constipated populations with slower transit times (Dimidi et al., 2014), the inclusion of participants with diarrhoea-predominant dysfunction (faster transit time) may have made the GTTs too variable, potentially making it difficult to identify a response in this domain.

The dairy matrix refers to the complex interplay of nutrients within the physical structure of a dairy food and plays a crucial role in influencing the digestion and bioavailability of nutrients and bioactive compounds (Feeney et al., 2018; Unger et al., 2023; O’Connor et al., 2024; Rooney et al., 2026). This structure can significantly enhance the stability and functionality of potentially postbiotic compounds, such as microbial metabolites or cell wall components, by protecting them from degradation in the gastrointestinal tract and supporting their delivery to target sites in the body (Aguilera, 2019). The dairy matrix has been shown to play a role in the neutral or beneficial effects of dairy on cardiometabolic health (Feeney et al., 2018; O’Connor et al., 2024; Rooney et al., 2025d), and the “matrix effect” may extend to gastrointestinal health (Ní Chonnacháin et al., 2024; Ní Chonnacháin et al., 2026). In the current study, participants consumed either a fermented SMP, a non-fermented SMP, or energy-matched, non-dairy maltodextrin, which were reconstituted with water prior to ingestion. Unlike whole dairy products such as milk, yoghurt, or cheese, these powders lack the original physical dairy food structure encapsulating the nutritional components (i.e., the dairy matrix), which could limit their potential to confer gut health benefits (Aguilera, 2019). As a result, despite containing potentially beneficial bioactive compounds, these reconstituted powders may not deliver the same health effects as whole fermented dairy foods (Unger et al., 2023). This has been previously demonstrated in similar studies, where the beneficial efficacy of nutritional components in fermented dairy foods, notably probiotic strains, can be dependent on their delivery matrix (Ní Chonnacháin et al., 2024). For example, Lee et al. found that when *Lacticaseibacillus casei* was administered in yoghurt or tablet form, a beneficial gastrointestinal response was only observed in the yoghurt group (Lee et al., 2015). Thus, considering our findings, perhaps the efficacy of the nutritional bioactives and fermented properties may be enhanced in a liquid milk matrix, rather than a reconstituted powder matrix. However, to further understand the dairy matrix effect in this context it would be useful to investigate the effects of fermented SMPs compared to fermented skimmed milk (liquid milk matrix) on gastrointestinal health parameters.

The present study had several strengths including randomised treatment allocation by an independent researcher not involved in the study, the double-blind study design, and the evaluation of dietary intake during the study period. While previous studies have investigated the effects of fermented dairy products on gastrointestinal health in both healthy (Matsumoto et al., 2010; Gomi et al., 2018) and IBS (Simrén et al., 2010; Šmid et al., 2016) subjects, relatively few have considered recurrent mild-to-moderate digestive issues or discomfort in otherwise healthy populations. This study also had some limitations, including a high rate of attrition. Of the 146 participants randomised to treatment, only 89 (61%) completed the entire study of which 84 (94.4%) provided data for the primary outcome at both Week 0 and Week 8. This was likely a result of the relatively high participant burden with five study-site visits and repeated questionnaires. Antibiotic use during the study period was another primary reason for participant attrition. The authors acknowledge that the unequal participant allocation is not a commonly employed approach; however, a statistician was consulted and equal sample size is not an assumption for ANOVA. The use of unequal randomisation ratios has been debated elsewhere (Dumville et al., 2006). Despite rigorous screening of potential participants to ensure all participants had gastrointestinal symptoms, the cohort studied was a generally healthy group of individuals with less severe symptoms than anticipated. As a result, there may have been limited potential for improvement in outcome measures and limiting the ability to observe an effect of the treatment. Furthermore, as this was a non-clinical cohort with self-reported symptoms, the population may have greater homogeneity which may reduce the interpretability of intervention effects. The authors also note the exploratory nature of stratification by symptom severity approach and as such, limit the ability to support efficacy claims for any of the treatments.

This RCT found a dairy powder fermented with *L. delbrueckii* subsp. *Bulgaricus* to have limited effects on QoL in those with self-reported gastrointestinal symptoms; however, a non-fermented SMP demonstrated some promising beneficial effects. Further research in those with more severe symptoms and in other dairy products, perhaps those with a different dairy matrix, is warranted to further examine the effects of dairy, whether fermented or not, on gastrointestinal health in non-clinical cohorts.

## Supporting information

10.1017/gmb.2026.10031.sm001 Rooney et al. supplementary material Rooney et al. supplementary material

## Data Availability

Data described in the manuscript will not be made available, in line with current UCD HREC data storage and retention guidelines; the de-identified archived data are only accessible to study investigators and will not be accessible to others.

## Supplementary material

The supplementary material for this article can be found at http://doi.org/10.1017/gmb.2026.10031.

## Long descriptions

### Figure 1 Long description

The study design comprised a 4-week baseline period, 8-week intervention phase, a 4-week ‘washout’ period. Diagram shows the five time points for participant study visits, data collection and stool sample collection. Gut transit time, quality of life questionnaires, anthropometry and dietary information was collected.

Navigate back to Figure 1.

### Figure 2 Long description

At enrolment, 3,020 were assessed for eligibility, with 2,874 excluded as not meeting inclusion criteria, lost to follow-up or declined to participate. A total of 146 were randomised to received 20 g/day of either fermented skimmed milk powder (87 participants), skimmed milk powder (21 participants) or maltodextrin (28 participants). Five visits were conducted. At the end, a total of 90 participants completed both the pre and post intervention appointments.

Navigate back to Figure 2.

### Figure 3 Long description

In FDDQoL there were significant differences between SMP and maltodextrin for anxiety, discomfort and global scores, and between FSMP and SMP for discomfort and global scores, where SMP demonstrated significant imrpovements compared to other treatments. In PACQoL SMP was shown to improve psychosocial discomfort compared to both FSMP and maltodextrin.

Navigate back to Figure 3.

### Figure 4 Long description

Two bar charts presenting each group at pre and post intervention (top graph) and response to intervention (bottom graph). No differences between groups or timepoints were observed for gut transit time.

Navigate back to Figure 4.

### Table 1. Long description

The table is divided into two primary sections.

1. Demographics and anthropometry:

- Age (years): Total 34.9 plus or minus 5.9. F S M P 35.6 plus or minus 6.4. S M P 34.0 plus or minus 5.1. Maltodextrin 34.2 plus or minus 5.3. P-value 0.575.

- Sex n (%): Male 32 (38.1), Female 52 (61.9). Distribution is similar across groups (P-value 0.821).

- Weight (kg): Baseline (Week 0) is approximately 77 to 78 kg across all groups. No significant change at Week 8.

- B M I (kg m super 2): Baseline is approximately 25 to 26 across all groups. No significant change at Week 8.

2. F D D Q o L Scores (Higher scores indicate better quality of life):

- Daily activities: F S M P and S M P groups showed significant within-group improvements (P < 0.001 and P = 0.006 respectively), while Maltodextrin did not (P = 0.341).

- Anxiety: S M P showed the largest delta increase (14.6 plus or minus 20.8) compared to Maltodextrin (0.9 plus or minus 18.3), with a significant delta P-value of 0.045.

- Diet: F S M P showed significant within-group improvement (P < 0.001).

- Discomfort: S M P showed a significant delta increase (15.5 plus or minus 18.3) compared to F S M P (6.3) and Maltodextrin (1.0), with a significant delta P-value of 0.005.

- Global score: Baseline was approximately 60 to 63. At Week 8, scores were F S M P 64.8, S M P 71.3, and Maltodextrin 63.4. The delta P-value across groups was 0.024, with S M P showing significant improvement over both other groups.

Navigate back to Table 1..
