# Supplementary material for: A Lactobacillus delbrueckii subsp. bulgaricus fermented skimmed milk powder has limited effects on gastrointestinal health in healthy individuals with mild-to-moderate gastrointestinal symptoms: results from a randomised controlled trial
Source: Gut Microbiome (Camb). 2026 Jul 31;7:e14. doi: 10.1017/gmb.2026.10031 (PMC13433340; doi:10.1017/gmb.2026.10031)
Supplement: Supplementary file 1 [file S2632289726100310sup001.docx]

**A *Lactobacillus delbrueckii* subsp. *bulgaricus*** **fermented skimmed milk powder has limited effects on gastrointestinal health in healthy individuals with mild-to-moderate gastrointestinal symptoms: Results from a randomised controlled trial**

Martina Rooney,†^1,2^ Clíona Ní Chonnacháin,†^1,2^ Bernadette Finnerty,^1,2,3^ Grace Bennett,^1,2^ Éimear Gregory, ^1,2^ Conor Carey,^1,3^ Monica A. Mechoud^1,4^, Ram Raj Panthi^1,4^, Harsh Mathur,^1,4^ Eoin Murphy,^1,4^ Tom Beresford,^1,4^ Paul D. Cotter,^1,4,5,6^ Alice Lucey,^1,3^ Emma L. Feeney.*^1,2^

**Supplemental Table 1:** Macronutrient composition of each study treatment per 20g daily dose.

**Supplemental Table 2.** Demographic and anthropometric data across groups at UCD and UCC sites.

**Supplemental Table 3.** Patient Assessment of Constipation Quality of Life (PACQoL) domain scores throughout study protocol (*n* 63).

**Supplemental Table 4.** Perceived Stress Questionnaire (PSQ) scores throughout the study protocol.

**Supplemental Table 5**. Subject Global Assessment (SGA) score, stool consistency and gas evacuations across the study protocol (*n* 63).

**Supplemental Table 6.** Gut transit time (GTT) across the study protocol.

**Supplemental Table 7.** Faecal calprotectin (FCP) across the study protocol.

**Supplemental Table 8.** Dietary Intake data throughout study protocol (*n* 53).

**Supplemental Table 9.** Quality of life scores in response to 8-week intervention with FSMP, SMP and maltodextrin, in males only (*n* 32).

**Supplemental Table 10.** Quality of life scores in response to 8-week intervention with FSMP, SMP and maltodextrin, in females only (*n* 52).

**Supplemental Table 11.** Functional Digestive Disorders Quality of Life (FDDQoL) global scores, Perceived Stress Questionnaire (PSQ) Index and Patient Assessment of Constipation Quality of Life (PACQoL) total scores throughout the study protocol within baseline symptom severity subgroups.

**Supplemental Table 12.** Subject Global Assessment (SGA) score, stool consistency and gas evacuations across the study protocol within baseline symptom severity subgroups.

**Supplemental Table 13**. Gut Transit Time (GTT) across the study protocol within baseline symptom severity subgroups

**Supplemental Figure 1.** Organic acid composition of dairy-containing intervention powders tested.

**Supplemental Figure 2.** Faecal calprotectin (FCP).

**Supplemental Table 1:** Macronutrient composition of each study treatment per 20g daily dose.

|  | **FSMP** | **SMP** | **Maltodextrin** |
| --- | --- | --- | --- |
| Energy (kcal) | 78 | 69.6 | 77 |
| Carbohydrate (g) | 10.5 | 10.6 | 19 |
| Fat (g) | 0 | 0 | 0 |
| Protein (g) | 7.2 | 7.2 | 0 |

FSMP, fermented skimmed milk powder; SMP, skimmed milk powder.

**Supplemental Table 2.** Demographic and anthropometric data across groups at UCD and UCC sites.

|  | **Total** | **FSMP** | **SMP** | **Maltodextrin** | ***P***  **Wk 0^a^** | ***P* Δ^b^** |
| --- | --- | --- | --- | --- | --- | --- |
| **UCD** | **(n 60)** | **(n 33)** | **(n 14)** | **(n 13)** |  |  |
| Age (years) | 34.6 ± 5.9 | 35.2 ± 6.2 | 33.1 ± 5.5 | 34.5 ± 5.5 | 0.609 | - |
| **Sex ^c^ *n* (%)** |  |  |  |  | 0.629 | - |
| Male | 26 (43.3) | 14 (42.4) | 5 (35.7) | 7 (53.8) |  |  |
| Female | 34 (56.7) | 19 (57.6) | 9 (64.3) | 6 (46.2) |  |  |
| **Weight (kg)** |  |  |  |  | 0.746 | 0.348 |
| Week 0 | 78.4 ± 16.2 | 79.6 ± 15.2 | 75.7 ± 20.0 | 78.0 ± 15.1 |  |  |
| Week 8 | 78.1 ± 15.7 | 79.7 ± 14.9 | 75.0 ± 18.8 | 77.5 ± 14.9 |  |  |
| **BMI (kg m^-2^)** |  |  |  |  | 0.388 | 0.389 |
| Week 0 | 26.0 ± 4.2 | 26.6 ± 4.5 | 25.4 ± 4.2 | 25.0 ± 3.1 |  |  |
| Week 8 | 25.9 ± 4.2 | 26.6 ± 4.6 | 25.2 ± 3.9 | 24.8 ± 3.1 |  |  |
| **UCC** | **(n 24)** | **(n 14)** | **(n 5)** | **(n 5)** |  |  |
| Age (years) | 35.8 ± 5.9 | 36.5 ± 7.0 | 36.4 ± 2.9 | 33.2 ± 5.1 | 0.594 | - |
| **Sex ^c^ *n* (%)** |  |  |  |  | 0.683 | - |
| Male | 6 (25.0) | 3 (21.4) | 2 (40.0) | 1 (20.0) |  |  |
| Female | 18 (75.0) | 11 (78.6) | 3 (60.0) | 4 (80.0) |  |  |
| **Weight (kg)** |  |  |  |  | 0.920 | 0.402 |
| Week 0 | 75.9 ± 16.4 | 74.9 ± 13.6 | 79.9 ± 24.8 | 74.7 ± 17.3 |  |  |
| Week 8 | 76.5 ± 17.2 | 75.1 ± 14.1 | 81.0 ± 26.1 | 76.0 ± 18.6 |  |  |
| **BMI (kg m^-2^)** |  |  |  |  | 0.841 | 0.427 |
| Week 0 | 26.6 ± 5.1 | 26.6 ± 4.4 | 27.7 ± 7.5 | 25.7 ± 5.1 |  |  |
| Week 8 | 26.9 ± 5.4 | 26.7 ± 4.6 | 28.1 ± 8.0 | 26.2 ± 5.4 |  |  |

Data presented as mean ± standard deviation, or *n* (%).

^a^ Differences across groups at Week 0 calculated with 1-factor ANOVA or Kruskal–Wallis nonparametric ANOVA where appropriate.

^b^ Differences across groups for delta values (Week 8–Week 0) calculated with 1-factor ANOVA or Kruskal–Wallis nonparametric ANOVA where appropriate.

^c^ Chi-square test.

**Supplemental Table 3.** Patient Assessment of Constipation Quality of Life (PACQoL) domain scores throughout study protocol (*n* 63).

|  | **Total**  **(*n* 63)** | **FSMP**  **(*n* 32)** | **SMP**  **(*n* 16)** | **Maltodextrin (*n* 15)** | ***P***  **Wk 0^a^** | ***P* Δ^b^** |
| --- | --- | --- | --- | --- | --- | --- |
| **Physical Discomfort** |  |  |  |  | 0.791 | 0.288 |
| Week 0 | 6.4 ± 4.0 | 6.5 ± 4.2 | 5.8 ± 3.3 | 6.9 ± 4.5 |  |  |
| Week 8 | 5.1 ± 3.7 | 5.1 ± 3.9 | 3.9 ± 3.5 | 6.3 ± 3.4 |  |  |
| Δ | -1.3 ± 2.9 | -1.4 ± 2.8 | -1.9 ± 3.1 | -0.6 ± 2.9 |  |  |
| *P* within group^c^ |  | 0.009 | 0.027 | 0.384 |  |  |
| **Psychosocial Discomfort** |  |  |  |  | 0.856 | 0.039^d^ |
| Week 0 | 9.1 ± 6.9 | 8.7 ± 6.6 | 9.0 ± 7.3 | 9.9 ± 7.4 |  |  |
| Week 8 | 7.0 ± 6.5 | 7.2 ± 6.4 | 4.9 ± 5.4 | 9.0 ± 7.4 |  |  |
| Δ | -2.1 ± 4.2 | -1.5 ± 2.8 | -4.1 ± 6.5 | -0.9 ± 2.7 |  |  |
| *P* within group^c^ |  | 0.003 | 0.025 | 0.208 |  |  |
| **Worries and Concerns** |  |  |  |  | 0.541 | 0.250 |
| Week 0 | 15.1 ± 9.9 | 15.4 ± 9.6 | 12.8 ± 9.2 | 16.9 ± 11.5 |  |  |
| Week 8 | 11.9 ± 9.8 | 12.8 ± 9.7 | 8.1 ± 8.0 | 13.9 ± 11.1 |  |  |
| Δ | -3.2 ± 6.2 | -2.6 ± 5.4 | -4.7 ± 8.1 | -3.0 ± 5.9 |  |  |
| *P* within group^c^ |  | 0.009 | 0.038 | 0.074 |  |  |
| **Satisfaction** |  |  |  |  | 0.343 | 0.397 |
| Week 0 | 11.5 ± 4.3 | 10.8 ± 4.7 | 11.9 ± 3.4 | 12.7 ± 4.4 |  |  |
| Week 8 | 9.9 ± 4.5 | 8.9 ± 4.5 | 10.1 ± 4.2 | 11.7 ± 4.4 |  |  |
| Δ | -1.6 ± 4.9 | -1.9 ± 5.2 | -1.8 ± 5.1 | -1.0 ± 4.3 |  |  |
| *P* within group^c^ |  | 0.058 | 0.189 | 0.416 |  |  |
| **Total Score** |  |  |  |  |  |  |
| Week 0 | 42.1 ± 22.0 | 41.3 ± 22.1 | 39.4 ± 19.7 | 46.4 ± 25.0 | 0.663 | 0.159 |
| Week 8 | 33.9 ± 21.3 | 33.9 ± 21.7 | 27.1 ± 17.6 | 40.9 ± 23.2 |  |  |
| Δ | -8.2 ± 12.8 | -7.4 ± 11.0 | -12.3 ± 17.1 | -5.5 ± 10.7 |  |  |
| *P* within group^c^ |  | <0.001 | 0.012 | 0.067 |  |  |

Data presented as mean ± standard deviation. A lower PACQoL score is associated with a better quality of life. Δ, delta values calculated as Week 8 minus Week 0. FSMP, fermented skimmed milk powder; SMP, skimmed milk powder.

^a^ Differences across groups for Week 0 calculated with 1-factor ANOVA or Kruskal–Wallis nonparametric ANOVA where appropriate.

^b^ Differences across groups for delta values (Week 8 – Week 0) calculated with general linear models controlling for study site, sex, age, BMI and Week 0 values. *Post hoc* analysis was conducted *via* LSD test.

^c^ Within-group differences calculated with Paired-samples T-Test.

^d^ Significant differences for Δ psychosocial discomfort between FSMP group and SMP (P=0.031) and between SMP and Maltodextrin (P=0.019).

**Supplemental Table 4.** Perceived Stress Questionnaire (PSQ) scores throughout the study protocol (*n* 84).

|  | **Total**  **(*n* 84)** | **FSMP**  **(*n* 47)** | **SMP**  **(*n* 19)** | **Maltodextrin (*n* 18)** | ***P***  **Wk 0^a^** | ***P* Δ^b^** |
| --- | --- | --- | --- | --- | --- | --- |
| **Harassment** |  |  |  |  | 0.219 | 0.366 |
| Week 0 | 9.5 ± 2.4 | 9.9 ± 2.1 | 8.7 ± 2.9 | 9.5 ± 2.4 |  |  |
| Week 8 | 9.0 ± 2.5 | 9.2 ± 2.1 | 8.1 ± 3.0 | 9.6 ± 2.7 |  |  |
| Δ | -0.5 ± 2.0 | -0.7 ± 2.2 | -0.6 ± 1.6 | 0.1 ± 1.8 |  |  |
| *P* within group^c^ |  | 0.043 | 0.126 | 0.899 |  |  |
| **Overload** |  |  |  |  | 0.080 | 0.176 |
| Week 0 | 8.5 ± 1.7 | 8.8 ± 1.6 | 7.8 ± 1.8 | 8.4 ± 1.6 |  |  |
| Week 8 | 8.1 ± 2.0 | 8.5 ± 1.8 | 7.0 ± 2.2 | 8.3 ± 1.9 |  |  |
| Δ | -0.4 ± 1.5 | -0.3 ± 1.5 | -0.8 ± 1.4 | -0.1 ± 1.7 |  |  |
| *P* within group^c^ |  | 0.157 | 0.028 | 0.782 |  |  |
| **Irritability** |  |  |  |  | 0.037^d^ | 0.411 |
| Week 0 | 4.5 ± 1.2 | 4.8 ± 0.8 | 4.2 ± 1.6 | 4.1 ± 1.2 |  |  |
| Week 8 | 4.2 ± 1.3 | 4.4 ± 1.3 | 3.8 ± 1.4 | 4.3 ± 1.4 |  |  |
| Δ | -0.3 ± 1.2 | -0.4 ± 1.2 | -0.4 ± 0.9 | 0.2 ± 1.2 |  |  |
| *P* within group^c^ |  | 0.027 | 0.090 | 0.579 |  |  |
| **Lack of Joy** |  |  |  |  | 0.033^e^ | 0.467 |
| Week 0 | 15.8 ± 3.4 | 16.5 ± 3.0 | 14.3 ± 4.1 | 15.2 ± 3.3 |  |  |
| Week 8 | 15.1 ± 3.9 | 15.7 ± 3.4 | 13.5 ± 5.0 | 15.3 ± 3.6 |  |  |
| Δ | -0.7 ± 2.4 | -0.8 ± 2.6 | -0.8 ± 2.2 | 0.1 ± 2.2 |  |  |
| *P* within group^c^ |  | 0.037 | 0.108 | 0.916 |  |  |
| **Fatigue** |  |  |  |  | 0.045^f^ | 0.051 |
| Week 0 | 8.4 ± 2.3 | 8.9 ± 2.0 | 7.5 ± 2.8 | 7.9 ± 2.5 |  |  |
| Week 8 | 7.7 ± 2.3 | 8.1 ± 2.0 | 6.3 ± 2.2 | 7.8 ± 2.8 |  |  |
| Δ | -0.7 ± 1.8 | -0.8 ± 1.9 | -1.2 ± 1.3 | -0.1 ± 1.8 |  |  |
| *P* within group^c^ | | 0.013 | <0.001 | 0.698 |  |  |
| **Worries** | |  |  |  | 0.011^g^ | 0.646 |
| Week 0 | 10.6 ± 2.8 | 11.3 ± 2.7 | 9.1 ± 3.2 | 10.4 ± 2.0 |  |  |
| Week 8 | 10.1 ± 2.8 | 10.6 ± 2.6 | 8.8 ± 3.0 | 10.2 ± 2.8 |  |  |
| Δ | -0.5 ± 2.3 | -0.7 ± 2.7 | -0.3 ± 1.5 | -0.2 ± 1.9 |  |  |
| *P* within group^c^ | | 0.073 | 0.380 | 0.717 |  |  |
| **Tension** | |  |  |  | 0.707 | 0.311 |
| Week 0 | 9.3 ± 2.6 | 9.4 ± 2.2 | 9.3 ± 3.2 | 8.9 ± 3.1 |  |  |
| Week 8 | 8.9 ± 2.6 | 8.9 ± 2.2 | 8.5 ± 3.0 | 9.1 ± 3.1 |  |  |
| Δ | -0.4 ± 1.8 | -0.5 ± 2.0 | -0.8 ± 1.3 | 0.2 ± 1.3 |  |  |
| *P* within group^c^ | | 0.091 | 0.026 | 0.726 |  |  |
| **PSQ Index** | |  |  |  | 0.051 | 0.150 |
| Week 0 | 0.41 ± 0.15 | 0.44 ± 0.12 | 0.34 ± 0.20 | 0.39 ± 0.16 |  |  |
| Week 8 | 0.37 ± 0.17 | 0.39 ± 0.13 | 0.29 ± 0.21 | 0.38 ± 0.17 |  |  |
| Δ | -0.04 ± 0.09 | -0.05 ± 0.10 | -0.05 ± 0.06 | 0.01 ± 0.08 |  |  |
| *P* within group^c^ |  | 0.003 | 0.001 | 0.879 |  |  |

Data presented as mean ± standard deviation. A higher PSQ score is associated with greater levels of stress.

Δ, delta values calculated as Week 8 minus Week 0. FSMP, fermented skimmed milk powder; SMP, skimmed milk powder.

^a^ Differences across groups for Week 0 calculated with 1-factor ANOVA or Kruskal–Wallis nonparametric ANOVA where appropriate.

^b^ Differences across groups for delta values (Week 8 – Week 0) calculated with general linear models controlling for study site, sex, age, BMI and Week 0 values. *Post hoc* analysis was conducted *via* LSD test.

^c^ Within-group differences calculated with Paired-samples T-Test.

^d^ Significant differences for irritability scores at Week 0 between FSMP and Maltodextrin (P=0.024)

^e^ Significant differences for lack of joy scores at Week 0 between FSMP and SMP (P=0.014)

^f^ Significant differences for fatigue scores at Week 0 between FSMP and SMP (P=0.024)

^g^ Significant differences for worries scores at Week 0 between FSMP and SMP (P = 0.003).

**Supplemental Table 5**. Subject Global Assessment (SGA) score, stool consistency and gas evacuations across the study protocol (*n* 63).

|  | **Total**  **(*n* 63)** | **FSMP**  **(*n* 32)** | **SMP**  **(*n* 16)** | **Maltodextrin (*n* 15)** | ***P***  **Wk 0^a^** | ***P* Δ^b^** |
| --- | --- | --- | --- | --- | --- | --- |
| **SGA^d^ score** |  |  |  |  | 0.897 | 0.679 |
| Week 0 | 2.3 ± 0.6 | 2.2 ± 0.6 | 2.3 ± 0.7 | 2.3 ± 0.7 |  |  |
| Week 8 | 2.9 ± 0.8 | 2.8 ± 0.8 | 3.1 ± 0.9 | 2.9 ± 0.8 |  |  |
| Δ | 0.6 ± 1.0 | 0.6 ± 0.9 | 0.8 ± 1.2 | 0.6 ± 0.7 |  |  |
| *P* within group^c^ | | <0.001 | 0.014 | 0.015 |  |  |
| **Stool consistency^e^ score** | |  |  |  | 0.886 | 0.140 |
| Week 0 | 3.7 ± 1.4 | 3.8 ± 1.4 | 3.6 ± 1.7 | 3.5 ± 1.2 |  |  |
| Week 8 | 3.7 ± 1.5 | 4.0 ± 1.5 | 3.8 ± 1.5 | 3.0 ± 1.5 |  |  |
| Δ | 0.0 ± 1.5 | 0.2 ± 1.5 | 0.2 ± 1.7 | -0.5 ± 1.2 |  |  |
| *P* within group^c^ | | 0.567 | 0.774 | 0.104 |  |  |
| **Gas evacuations^f^** | |  |  |  | 0.779 | 0.112 |
| Week 0 | 14.6 ± 10.4 | 13.1 ± 7.7 | 16.0 ± 11.6 | 16.2 ± 13.7 |  |  |
| Week 8 | 13.3 ± 7.8 | 10.9 ± 5.7 | 15.8 ± 9.7 | 15.7 ± 8.3 |  |  |
| Δ | -1.3 ± 9.2 | -2.2 ± 6.0 | -0.2 ± 8.0 | -0.5 ± 14.5 |  |  |
| *P* within group^c^ |  | 0.054 | 0.921 | 0.903 |  |  |

Data presented as mean ± standard deviation.

Δ, delta values calculated as Week 8 minus Week 0. FSMP, fermented skimmed milk powder; SMP, skimmed milk powder.

^a^ Differences across groups for Week 0 calculated with 1-factor ANOVA or Kruskal–Wallis nonparametric ANOVA where appropriate.

^b^ Differences across groups for delta values (Week 8 – Week 0) calculated with general linear models controlling for study site, sex, age, BMI and Week 0 values.

^c^ Within-group differences calculated with Paired-samples T-Test.

^d^ Subject Global Assessment (SGA) (Müller-Lissner *et al*., 2003). An increase in a participant's SGA score represents an increased feeling of relief from gut symptoms or discomfort.

^e^ Stool consistency assessed according to the Bristol Stool Form Chart (BSFC) where lower scores indicate harder stools, on a scale of 1-7 (Lewis and Heaton, 1997).

^f^ Total *n* 58, FSMP *n* 29, SMP *n* 14, Maltodextrin *n* 15.

**Supplemental Table 6.** Gut transit time (GTT) across the study protocol.

|  | **GTT (hh:mm)** | | | | | |  |  |  |
| --- | --- | --- | --- | --- | --- | --- | --- | --- | --- |
|  | **Week 0** | | **Week 8** | | **Δ** | |  |  |  |
|  | **Median** | **IQR** | **Median** | **IQR** | **Median** | **IQR** | **P^a^** | **P^b^** | **P^c^** |
| **Total (n 68)** | 22:27 | 15:30 - 42:26 | 25:00 | 16:30 - 40:07 | -00:30 | -9:41 - 11:22 |  | 0.322 | 0.107 |
| **FMSP (n 37)** | 21:15 | 15:32 - 41:37 | 25:05 | 15:42 - 42:30 | 01:00 | -8:23 - 12:10 | 0.512 |  |  |
| **SMP (n 17)** | 33:30 | 17:40 - 52:57 | 25:00 | 20:24 - 28:49 | -07:29 | -17:05 - 4:55 | 0.113 |  |  |
| **Maltodextrin (n 14)** | 20:56 | 14:13 - 30:23 | 24:55 | 15:22 - 41:07 | 00:40 | -09:08 - 18:07 | 0.730 |  |  |

Data presented as medians and interquartile ranges (Q1 – Q3).

Δ, delta values calculated as Week 8 minus Week 0. FSMP, fermented skimmed milk powder; SMP, skimmed milk powder; hh:mm hours and minutes; IQR interquartile range.

p^a^ Within group differences (Week 0 vs Week 8) calculated using Wilcoxon signed-rank tests.

p^b^ Differences across groups for Week 0 calculated using Quade’s ANCOVA controlling for sex and body mass index.

p^c^ Differences across groups for delta values (Week 8 – Week 0) calculated using Quade’s ANCOVA controlling for sex and body mass index.

**Supplemental Table 7.** Faecal calprotectin (FCP) across the study protocol.

|  | **FCP (µg/g)** | | | | | | | |  |  | |
| --- | --- | --- | --- | --- | --- | --- | --- | --- | --- | --- | --- |
|  | **Total (n 72)** | | **FSMP (n 41)** | | **SMP (n 16)** | | **Maltodextrin (n 15)** | |  |  |  |
|  | **Median** | **IQR** | **Median** | **IQR** | **Median** | **IQR** | **Median** | **IQR** | **P Visit 1** | **PΔ** |  |
| **Timepoint** |  |  |  |  |  |  |  |  |  |  |  |
| Study Visit 1 | 10.00 | 4.00 - 27.75 | 12.00 | 12.00 - 26.00 | 8.50 | 4.00 - 26.00 | 10.00 | 4.00 - 15.00 | 0.714 | 0.639 |  |
| Study Visit 3 | 13.50 | 5.00 - 35.50 | 13.00 | 13.00 - 36.50 | 15.00 | 5.00 - 37.25 | 14.00 | 5.00 - 30.00 |  |  |  |
| Study Visit 5 | 13.50 | 13.50 - 21.75 | 14.00 | 14.00 - 21.50 | 12.50 | 12.50 - 24.25 | 12.00 | 12.00 - 20.00 |  |  |  |

Data presented as medians and interquartile ranges (Q1 – Q3).

Δ, delta values calculated as Visit 5 minus Visit 1. FCP, faecal calprotectin; FSMP, fermented skimmed milk powder; SMP, skimmed milk powder; IQR interquartile range.

Study Visits 1, 3 and 5 refer to weeks -4, 4 and 12, respectively, in the intervention timeline.

Differences across groups at Visit 1 calculated using a Kruskal-Wallis test.

Differences across groups for delta values (Visit 5 – Visit 1) calculated using Quade’s ANCOVA controlling for sex and site.

**Supplemental Table 8.** Dietary Intake data throughout study protocol (*n* 53).

|  | **Total** | **FSMP** | **SMP** | **Maltodextrin** | ***P* Wk 0^a^** | | ***P*Δ^b^** |
| --- | --- | --- | --- | --- | --- | --- | --- |
|  | **(n 53)** | **(n 28)** | **(n 11)** | **(n 14)** |  | |  |
| **Energy (kcal/day)** |  |  |  |  | 0.301 | | 0.514 |
| Week 0 | 1787 ± 604 | 1716 ± 599 | 1927 ± 663 | 1818 ± 591 |  | |  |
| Week 8 | 1844 ± 761 | 1715 ± 734 | 2138 ± 697 | 1871 ± 844 |  | |  |
| **Protein (% TE)** |  |  |  |  | 0.795 | | 0.507 |
| Week 0 | 17.3 ± 5.5 | 17.2 ± 5.7 | 16.6 ± 4.9 | 18.1 ± 6.0 |  | |  |
| Week 8 | 18.6 ± 5.0 | 18.1 ± 4.4 | 17.8 ± 4.7 | 20.1 ± 6.3 |  | |  |
| **Fat (% TE)** |  |  |  |  | 0.491 | 0.020**^c^** | |
| Week 0 | 38.2 ± 8.4 | 37.2 ± 8.8 | 38.0 ± 6.4 | 40.5 ± 9.2 |  | |  |
| Week 8 | 37.5 ± 7.4 | 35.3 ± 8.0 | 43.0 ± 4.6 | 37.6 ± 6.1 |  | |  |
| **CHO (% TE)** |  |  |  |  | 0.281 | | 0.174 |
| Week 0 | 45.7 ± 9.3 | 47.1 ± 10.0 | 46.4 ± 7.4 | 42.3 ± 8.7 |  | |  |
| Week 8 | 45.0 ± 10.0 | 47.6 ± 9.9 | 41.4 ± 8.2 | 42.9 ± 10.8 |  | |  |
| **Fibre (g per day)** |  |  |  |  | 0.708 | | 0.230 |
| Week 0 | 15.9 ± 8.3 | 15.6 ± 8.8 | 16.9 ± 6.7 | 15.9 ± 8.7 |  | |  |
| Week 8 | 16.1 ± 7.5 | 15.7 ± 7.0 | 19.5 ± 8.7 | 14.4 ± 7.0 |  | |  |
| **Sugars (% TE)** |  |  |  |  | 0.705 | | 0.383 |
| Week 0 | 17.8 ± 5.9 | 18.2 ± 7.0 | 16.5 ± 4.4 | 18.1 ± 4.5 |  | |  |
| Week 8 | 16.2 ± 8.0 | 17.0 ± 9.8 | 13.6 ± 5.2 | 16.5 ± 5.1 |  | |  |
| **Iron (mg/day)** |  |  |  |  | 0.634 | | 0.518 |
| Week 0 | 13.2 ± 11.1 | 13.1 ± 9.2 | 9.4 ± 3.3 | 16.2 ± 17.0 |  | |  |
| Week 8 | 16.2 ± 29.2 | 20.2 ± 39.8 | 12.3 ± 5.4 | 11.2 ± 4.8 |  | |  |
| **Calcium (mg/day)** | |  |  |  | 0.822 | | 0.569 |
| Week 0 | 754.2 ± 324.3 | 743.6 ± 322.5 | 731.6 ± 401.5 | 793.1 ± 279.8 |  | |  |
| Week 8 | 809.2 ± 411.0 | 780.5 ± 399.8 | 918.6 ± 570.3 | 780.4 ± 284.3 |  | |  |

Data presented as mean ± standard deviation.

Δ, delta values calculated as Week 8 minus Week 0. CHO, carbohydrate; FSMP, fermented skimmed milk powder; kcal, kilocalories. SMP, skimmed milk powder. TE, total energy

^a^ Differences across groups at Week 0 calculated with 1-factor ANOVA or Kruskal–Wallis nonparametric ANOVA where appropriate.

^b^ Differences across groups for delta values (Week 8 – Week 0) calculated with general linear models controlling for study site, sex, age, BMI and Week 0 values.

**^c^** Significant differences for Δ fat intake as a percentage of energy intake between FSMP group and SMP group (P=0.005).

**Supplemental Table 9.** Quality of life scores in response to 8-week intervention with FSMP, SMP and maltodextrin, in males only (*n* 32).

|  | **FSMP**  **(*n* 17)** | **SMP**  **(*n* 7)** | **Maltodextrin**  **(*n* 8)** | ***P***  **Wk 0^a^** | ***P* Δ^b^** |
| --- | --- | --- | --- | --- | --- |
| **FDDQoL Global Domain** | |  |  | 0.840 | 0.384 |
| Week 0 | 63.18 ± 14.04 | 61.70 ± 18.39 | 60.11 ± 11.99 |  |  |
| Week 8 | 66.62 ± 11.49 | 70.41 ± 16.03 | 65.45 ± 8.75 |  |  |
| Δ | 2.89 ± 9.65 | 8.71 ±9.87 | 5.34 ± 7.12 |  |  |
| *P* within group^c^ | 0.233 | 0.058 | 0.072 |  |  |
| **PSQ Index Score** |  |  |  | 0.569 | 0.128 |
| Week 0 | 0.46 ± 0.11 | 0.39 ± 0.21 | 0.41 ± 0.17 |  |  |
| Week 8 | 0.44 ± 0.13 | 0.35 ± 0.19 | 0.45 ± 0.19 |  |  |
| Δ | -0.02 ± 0.09 | -0.05 ± 0.08 | 0.04 ± 0.05 |  |  |
| *P* within group^c^ | 0.470 | 0.208 | 0.052 |  |  |
| **PACQoL Total Score** |  |  |  | 0.849 | 0.406 |
| Week 0 | 36.5 ± 23.4 | 41.7 ± 26.4 | 42.8 ± 21.0 |  |  |
| Week 8 | 34.5 ± 27.7 | 29.3 ± 26.3 | 32.0 ± 22.7 |  |  |
| Δ | -2.0 ± 10.2 | -12.3 ± 18.3 | -8.8 ± 10.9 |  |  |
| *P* within group^c^ | 0.551 | 0.160 | 0.104 |  |  |
| **SGA** |  |  |  | 0.622 | 0.777 |
| Week 0 | 2.30 ± 0.67 | 2.33 ± 0.82 | 2.67 ± 0.82 |  |  |
| Week 8 | 2.80 ± 0.63 | 2.83 ± 1.7 | 3.17 ± 0.75 |  |  |
| Δ | 0.50 ± 0.85 | 0.50 ± 1.38 | 0.50 ± 0.84 |  |  |
| *P* within group^c^ | 0.096 | 0.415 | 0.203 |  |  |
| **BSFC** |  |  |  | 0.674 | 0.722 |
| Week 0 | 4.5 ± 1.5 | 4.0 ± 1.9 | 3.8 ± 1.1 |  |  |
| Week 8 | 4.3 ± 1.3 | 4.0 ± 0.9 | 3.6 ± 1.5 |  |  |
| Δ | -0.2 ± 2.1 | 0.0 ± 1.4 | -0.2 ± 1.1 |  |  |
| *P* within group^c^ |  |  |  |  |  |
| **GTT (hh:mm)** |  |  |  | 0.334 | 0.800 |
| Week 0 | 23:40 ± 13:00 | 29:42 ± 19:38 | 15:41 ± 7:36 |  |  |
| Week 8 | 25:59 ± 15:33 | 27:43 ± 16:13 | 16:17 ± 8:32 |  |  |
| Δ | 2:19 ± 15:53 | -1:59 ± 6:39 | 0:36 ± 2:33 |  |  |
| *P* within group^c^ | 0.595 | 0.499 | 0.668 |  |  |
| **Gas evacuations** |  |  |  | 0.889 | 0.407 |
| Week 0 | 13.8 ± 8.0 | 14.4 ± 7.3 | 15.8 ± 8.8 |  |  |
| Week 8 | 32.6 ± 16.4 | 38.3 ± 19.7 | 45.0 ± 26.1 |  |  |
| Δ | 18.8 ± 10.1 | 23.8 ± 16.0 | 29.2 ± 20.8 |  |  |
| *P* within group^c^ | <0.001 | 0.015 | 0.019 |  |  |

Data presented as mean ± standard deviation. A higher score in FDDQoL, PSQ and SGA is associated with a better quality of life. A lower PACQoL score is associated with a better QoL.

Abbreviations: Δ, delta values calculated as Week 8 minus Week 0; BSFC, Bristol Stool Form Chart; FDDQoL, Functional Digestive Disorders Quality of Life; FSMP, fermented skimmed milk powder; PACQoL, Patient Assessment of Constipation Quality of Life; PSQ, Perceived Stress Questionnaire; SGA, Subject Global Assessment of Relief; SMP, skimmed milk powder.

^a^ Differences across groups for Week 0 calculated with 1-factor ANOVA or Kruskal–Wallis nonparametric ANOVA where appropriate.

^b^ Differences across groups for delta values (Week 8 – Week 0) calculated with general linear models controlling for study site, age, BMI and Week 0 values. *Post hoc* analysis was conducted *via* LSD test.

^c^ Within-group differences calculated with Paired-samples T-Test.

**Supplemental Table 10.** Quality of life scores in response to 8-week intervention with FSMP, SMP and maltodextrin, in females only (*n* 52).

|  | **FSMP**  **(*n* 30)** | **SMP**  **(*n* 12)** | **Maltodextrin**  **(*n* 10)** | ***P***  **Wk 0^a^** | ***P* Δ^b^** |
| --- | --- | --- | --- | --- | --- |
| **FDDQoL Global Domain** | |  |  | 0.371 | 0.033^d^ |
| Week 0 | 57.82 ± 14.39 | 63.58 ± 13.57 | 63.10 ± 12.59 |  |  |
| Week 8 | 63.90 ± 12.80 | 71.79 ± 9.11 | 61.72 ± 15.94 |  |  |
| Δ | 6.07 ± 8.02 | 8.21 ± 11.57 | -1.38 ± 7.85 |  |  |
| *P* within group^c^ | <0.001 | 0.032 | 0.592 |  |  |
| **PSQ Index Score** |  |  |  | 0.076 | 0.206 |
| Week 0 | 0.44 ± 0.12 | 0.38 ± 0.21 | 0.32 ± 0.15 |  |  |
| Week 8 | 0.38 ± 0.12 | 0.31 ± 0.25 | 0.33 ± 0.13 |  |  |
| Δ | -0.07 ± 0.10 | -0.07 ± 0.06 | 0.01 ± 0.09 |  |  |
| *P* within group^c^ | 0.002 | 0.010 | 0.771 |  |  |
| **PACQoL Total Score** |  |  |  | 0.577 | 0.057 |
| Week 0 | 42.8 ± 21.0 | 36.9 ± 17.9 | 48.8 ± 28.3 |  |  |
| Week 8 | 33.7 ± 20.1 | 24.1 ± 11.8 | 45.6 ! 23.6 |  |  |
| Δ | -9.1 ± 10.3 | -12.8 ± 19.5 | -3.2 ± 10.5 |  |  |
| *P* within group^c^ | <0.001 | 0.108 | 0.385 |  |  |
| **SGA** |  |  |  | 0.274 | 0.379 |
| Week 0 | 2.25 ± 0.44 | 2.25 ± 0.46 | 2.11 ± 0.60 |  |  |
| Week 8 | 2.80 ± 0.89 | 3.25 ± 0.89 | 2.67 ± 0.87 |  |  |
| Δ | 0.55 ± 0.94 | 1.00 ± 1.07 | 0.56 ± 0.73 |  |  |
| *P* within group^c^ | 0.017 | 0.033 | 0.051 |  |  |
| **BSFC** |  |  |  | 0.569 | 0.167 |
| Week 0 | 3.7 ± 1.5 | 3.1 ± 1.5 | 3.4 ± 1.4 |  |  |
| Week 8 | 3.8 ± 1.6 | 3.6 ± 2.0 | 2.7 ± 1.6 |  |  |
| Δ | 0.1 ± 1.2 | 0.5 ± 2.0 | -0.8 ± 1.3 |  |  |
| *P* within group^c^ |  |  |  |  |  |
| **GTT (hh:mm)** |  |  |  | 0.688 | 0.209 |
| Week 0 | 33:50 ± 28:29 | 39:59 ± 26:35 | 30:04 ± 21:53 |  |  |
| Week 8 | 36:26 ± 29:38 | 26:00 ± 10:34 | 29:33 ± 15:12 |  |  |
| Δ | 2:35 ± 19:05 | -13:58 ± 25:09 | -0.31 ± 27:07 |  |  |
| *P* within group^c^ | 0.521 | 0.095 | 0.953 |  |  |
| **Gas evacuations** |  |  |  | 0.285 | 0.131 |
| Week 0 | 12.1 ± 7.2 | 20.1 ± 14.5 | 16.4 ± 16.7 |  |  |
| Week 8 | 33.4 ± 18.3 | 60.4 ± 31.3 | 48.9 ± 25.6 |  |  |
| Δ | 21.3 ± 14.8 | 40.3 ± 24.6 | 32.4 ± 29.9 |  |  |
| *P* within group^c^ | <0.001 | 0.005 | 0.012 |  |  |

Data presented as mean ± standard deviation. A higher score in FDDQoL, PSQ and SGA is associated with a better quality of life. A lower PACQoL score is associated with a better QoL.

Abbreviations: Δ, delta values calculated as Week 8 minus Week 0; BSFC, Bristol Stool Form Chart; FDDQoL, Functional Digestive Disorders Quality of Life; FSMP, fermented skimmed milk powder; PACQoL, Patient Assessment of Constipation Quality of Life; PSQ, Perceived Stress Questionnaire; SGA, Subject Global Assessment of Relief; SMP, skimmed milk powder.

^a^ Differences across groups for Week 0 calculated with 1-factor ANOVA or Kruskal–Wallis nonparametric ANOVA where appropriate.

^b^ Differences across groups for delta values (Week 8 – Week 0) calculated with general linear models controlling for study site, age, BMI and Week 0 values. *Post hoc* analysis was conducted *via* LSD test.

^c^ Within-group differences calculated with Paired-samples T-Test.

^d^ Significant difference in response to intervention between SMP and maltodextrin (P=0.010)

**Supplemental Table 11.** Functional Digestive Disorders Quality of Life (FDDQoL) global scores, Perceived Stress Questionnaire (PSQ) Index and Patient Assessment of Constipation Quality of Life (PACQoL) total scores throughout the study protocol within baseline symptom severity subgroups.

|  | **Those with severe symptoms (*n* 42)** | | | | | **Those with moderate symptoms (*n* 42)** | | | | | |  |
| --- | --- | --- | --- | --- | --- | --- | --- | --- | --- | --- | --- | --- |
|  | **FSMP**  (*n* 26) | **SMP**  (*n* 7) | **Maltodextrin** (*n* 9) | ***P* Wk0 ^a^** | ***P*Δ ^b^** | | **FSMP**  (*n* 21) | **SMP**  (*n* 12) | **Maltodextrin** (*n* 9) | ***P* Wk0 ^a^** | ***P*Δ ^b^** | |
| **FDDQoL** |  |  |  | 0.403 | 0.054 | |  |  |  | 0.848 | 0.297 | |
| Week 0 | 50.2 ± 9.7 | 47.0 ± 6.5 | 53.4 ± 9.9 |  |  | | 72.1 ± 9.0 | 72.2 ± 9.6 | 70.2 ± 7.2 |  |  | |
| Week 8 | 58.1 ± 11.5 | 64.2 ± 11.0 | 57.1 ± 12.6 |  |  | | 73.3 ± 6.8 | 75.4 ± 10.3 | 69.7 ± 10.7 |  |  | |
| *P* within group^c^ | <0.001 | <0.001 | 0.222 |  |  | | 0.441 | 0.230 | 0.843 |  |  | |
| **PSQ** |  |  |  | 0.085 | 0.050^e^ | |  |  |  | 0.183 | 0.876 | |
| Week 0 | 0.47 ± 0.10 | 0.43 ± 0.17 | 0.37 ± 0.13 |  |  | | 0.40 ± 0.13 | 0.29 ± 0.20 | 0.40 ± 0.18 |  |  | |
| Week 8 | 0.41 ± 0.14 | 0.33 ± 0.19 | 0.39 ± 0.13 |  |  | | 0.37 ± 0.12 | 0.26 ± 0.22 | 0.38 ± 0.22 |  |  | |
| *P* within group^c^ | 0.007 | 0.004 | 0.269 |  |  | | 0.158 | 0.085 | 0.434 |  |  | |
| **PACQoL^d^** |  |  |  | 0.901 | 0.019^f^ | |  |  |  | 0.398 | 0.497 | |
| Week 0 | 54.2 ± 19.3 | 50.6 ± 25.0 | 56.6 ± 29.3 |  |  | | 26.7 ± 14.9 | 34.4 ± 15.6 | 34.7 ± 12.4 |  |  | |
| Week 8 | 45.4 ± 21.6 | 25.4 ± 20.2 | 49.6 ± 24.4 |  |  | | 21.0 ± 13.3 | 27.9 ± 17.2 | 31.0 ± 18.5 |  |  | |
| *P* within group^c^ | 0.008 | 0.086 | 0.036 |  |  | | 0.043 | 0.031 | 0.503 |  |  | |

Data presented as mean ± standard deviation.

Δ, delta values calculated as Week 8 minus Week 0. FSMP, fermented skimmed milk powder; SMP, skimmed milk powder.

^a^ Differences across groups for Week 0 calculated with 1-factor ANOVA or Kruskal–Wallis nonparametric ANOVA where appropriate.

^b^ Differences across groups for delta values (Week 8 – Week 0) calculated with general linear models controlling for study site, sex, age, BMI and Week 0 values.

^c^ Within-group differences calculated with Paired-samples T-Test.

^d^ Those with severe symptoms (*n* 30), FSMP *n* 17, SMP *n* 5, Maltodextrin *n* 8; Those with moderate symptoms (*n* 33), FSMP *n* 15, SMP *n* 11, Maltodextrin *n* 7.

^e^ Significant differences for Δ PSQ Index between SMP group and Maltodextrin group (P = 0.017).

^f^ Significant differences for Δ PACQoL Total Score between FSMP group and SMP group (P = 0.007) and SMP group and Maltodextrin group (P = 0.014).

**Supplemental Table 12.** Subject Global Assessment (SGA) score, stool consistency and gas evacuations across the study protocol within baseline symptom severity subgroups.

|  | **Those with severe symptoms (*n* 30)** | | | | | **Those with moderate symptoms (*n* 33)** | | | | |
| --- | --- | --- | --- | --- | --- | --- | --- | --- | --- | --- |
|  | **FSMP**  (*n* 17) | **SMP**  (*n* 5) | **Maltodextrin** (*n* 8) | ***P* Wk0 ^a^** | ***P*Δ ^b^** | **FSMP**  (*n* 15) | **SMP**  (*n* 11) | **Maltodextrin** (*n* 7) | ***P* Wk0 ^a^** | ***P*Δ ^b^** |
| **SGA ^d^** |  |  |  | 0.895 | 0.487 |  |  |  | 0.864 | 0.791 |
| Week 0 | 2.1 ± 0.6 | 2.0 ± 0.0 | 2.1 ± 0.6 |  |  | 2.4 ± 0.5 | 2.4 ± 0.8 | 2.6 ± 0.8 |  |  |
| Week 8 | 2.9 ± 0.8 | 3.2 ± 0.8 | 2.9 ± 1.0 |  |  | 2.8 ± 0.9 | 3.0 ± 1.0 | 2.9 ± 0.7 |  |  |
| *P* within group^c^ | 0.003 | 0.033 | 0.020 |  |  | 0.111 | 0.132 | 0.356 |  |  |
| **Stool Consistency ^e^ score** |  |  |  | 0.327 | 0.05^g^ |  |  |  | 0.476 | 0.633 |
| Week 0 | 4.1 ± 1.4 | 3.4 ± 2.5 | 3.1 ± 1.4 |  |  | 3.5 ± 1.4 | 3.7 ± 1.3 | 4.0 ± 1.0 |  |  |
| Week 8 | 4.4 ± 1.5 | 3.4 ± 1.1 | 2.6 ± 1.8 |  |  | 3.5 ± 1.2 | 3.9 ± 1.6 | 3.4 ± 1.1 |  |  |
| *P* within group^c^ | 0.351 | 1.000 | 0.227 |  |  | 1.000 | 0.749 | 0.321 |  |  |
| **Gas Evacuations^f^** |  |  |  | 0.078 | 0.025^h^ |  |  |  | 0.745 | 0.257 |
| Week 0 | 14.1 ± 7.9 | 20.0 ± 2.2 | 19.1 ± 18.7 |  |  | 12.1 ± 7.7 | 13.8 ± 14.1 | 13.6 ± 7.6 |  |  |
| Week 8 | 11.3 ± 4.9 | 20.4 ± 9.2 | 15.3 ± 5.0 |  |  | 10.5 ± 6.7 | 13.2 ± 9.4 | 16.1 ± 10.8 |  |  |
| *P* within group^c^ | 0.085 | 0.929 | 0.602 |  |  | 0.348 | 0.832 | 0.513 |  |  |

Data presented as mean ± standard deviation.

Δ, delta values calculated as Week 8 minus Week 0. FSMP, fermented skimmed milk powder; SMP, skimmed milk powder.

^a^ Differences across groups for Week 0 calculated with 1-factor ANOVA or Kruskal–Wallis nonparametric ANOVA where appropriate.

^b^ Differences across groups for delta values (Week 8 – Week 0) calculated with general linear models controlling for study site, sex, age, BMI and Week 0 values.

^c^ Within-group differences calculated with Paired-samples T-Test.

^d^ Subject Global Assessment (SGA) (Müller-Lissner *et al*., 2003). An increase in a participant's SGA score represents an increased feeling of relief from gut symptoms or discomfort.

^e^ Stool consistency assessed according to the Bristol Stool Form Chart (BSFC) where lower scores indicate harder stools, on a scale of 1-7 (Lewis and Heaton, 1997).

^f^ Those with severe symptoms (*n* 27), FSMP *n* 15, SMP *n* 5, Maltodextrin *n* 7; Those with moderate symptoms (*n* 31), FSMP *n* 14, SMP *n* 9, Maltodextrin *n* 8.

^g^ Significant differences for Δ stool consistency score between FSMP group and Maltodextrin group (P = 0.016).

^h^ Significant differences for Δ gas evacuations between FSMP group and SMP (P = 0.008).

**Supplemental Table 13**. Gut Transit Time (GTT) across the study protocol within baseline symptom severity subgroups

|  | **Those with severe symptoms (*n* 31)** | | | | | | **Those with moderate symptoms (*n* 35)** | | | | | |
| --- | --- | --- | --- | --- | --- | --- | --- | --- | --- | --- | --- | --- |
|  | **FSMP**  (*n* 18) | **SMP**  (*n* 6) | **Maltodextrin** (*n* 7) | ***P* Wk0 ^a^** | ***P*Δ ^c^** | **FSMP**  (*n* 17) | | **SMP**  (*n* 11) | **Maltodextrin** (*n* 7) | ***P* Wk0 ^a^** | ***P*Δ ^c^** |  |
| **GTT** |  |  |  | 0.675 | 0.714 |  | |  |  | 0.024^d^ | 0.203 |  |
| Week 0 | 36:27 ± 29:52 | 29:04 ± 20:13 | 36:18 ± 23:55 |  |  | 25:45 ± 15:15 | | 40:20 ± 26:12 | 15:36 ± 04:51 |  |  |  |
| Week 8 | 33:32 ± 30:43 | 28:28 ± 13:55 | 29:33 ± 13:25 |  |  | 33:27 ± 20:28 | | 25:36 ± 12:00 | 21:58 ±15:54 |  |  |  |
| *P* within group^c^ | 0.430 | 0.914 | 0.554 |  |  | 0.128 | | 0.063 | 0.271 |  |  |  |

Data presented as mean ± standard deviation.

Δ, delta values calculated as Week 8 minus Week 0. FSMP, fermented skimmed milk powder; SMP, skimmed milk powder.

^a^ Differences across groups for Week 0 calculated with 1-factor ANOVA or Kruskal–Wallis nonparametric ANOVA where appropriate.

^b^ Differences across groups for delta values (Week 8 – Week 0) calculated with general linear models controlling for study site, sex, age, BMI and Week 0 values.

^c^ Within-group differences calculated with Paired-samples T-Test.

^d^ Significant differences for gut transit time at Week 0 between SMP group and Maltodextrin group (P=0.006).

Organis Acid Concentration (mg/mL)

|  | FSMP | SMP |
| --- | --- | --- |
| Lactic acid | 6.35838 | 0 |
| Galactose | 1.10686 | 0 |
| Glucose | 2.40794 | 0 |
| Lactose | 8.03432 | 13.5439 |

**Supplemental Figure 1.** Organic acid composition of dairy-containing intervention powders tested. Concentrations of lactic acid, galactose, glucose and lactose of a *Lactobacillus delbrueckii* subsp. *bulgaricus* fermented skimmed milk powder (FSMP, green shaded bars) and a skimmed milk powder (SMP, blue shaded bars).

**Supplemental Figure 2.** Faecal calprotectin (FCP).

Faecal calprotectin (µg/g) split by treatment group and timepoint (a) and Change (Δ) in faecal calprotectin (µg/g) across treatment groups between Visit 1 (Week -4) and Visit 5 (Week 12) (b).

Boxes represent interquartile ranges (Q3 – Q1) and horizontal lines within boxes represent medians.

Visits 1, 3 and 5 refer to Weeks -4, 4 and 12, respectively, in the intervention timeline

Faecal calprotectin (FCP); FSMP, fermented skimmed milk powder; SMP, skimmed milk powder
